# Systematic Identification of Chemical Components and Analysis of Major Constituents of *Verbena officinalis* L. Based on UHPLC–Q–Exactive–Orbitrap MS Combined with Feature-Based Molecular Networking and SIRIUS Strategy

**DOI:** 10.3390/molecules31132244

**Published:** 2026-06-25

**Authors:** Wenqing Xiao, Meng Li, Huibin Luo, Qiru Chen, Liangyin Shu, Liangjun Guan, Shunli Xiao

**Affiliations:** 1 School of Pharmaceutical Sciences, Hunan University of Medicine, Huaihua 418000, China; 19573970618@163.com (W.X.); m18890213996@163.com (M.L.); lhb202405@163.com (H.L.); 18821968491@163.com (Q.C.); shuliangyin@foxmail.com (L.S.); 2 School of Pharmacy, Shandong Second Medical University, Weifang 261000, China

**Keywords:** UHPLC–Q–Exactive–Orbitrap MS, Feature-Based Molecular Networking, *Verbena officinalis* L., SIRIUS, components

## Abstract

*Verbena officinalis* L., a classic medicinal plant of the Verbenaceae family with wide clinical applications, contains alkaloids and steroids with potent anti-inflammatory and anti-psoriatic activities. However, its whole-plant chemical composition and pharmacological material basis have not been systematically elucidated. Here, UHPLC–Q–Exactive–Orbitrap MS combined with FBMN and SIRIUS software was applied for comprehensive component analysis. A total of 126 constituents were annotated following MSI standards: 15 were unambiguously identified as MSI Level 1 using reference standards, 80 were tentatively assigned to MSI Level 2 via literature MS/MS data and GNPS spectral matching, and the remaining 31 were annotated as MSI Level 3 by in silico prediction with SIRIUS. Among them, 74 compounds were detected in this plant for the first time, and 15 were preliminarily regarded as putative novel candidate constituents. This integrated method shows better isomer resolution than traditional GNPS workflows, greatly improving the efficiency and accuracy of chemical profiling of medicinal plants.

## 1. Introduction

*Verbena* Linn. belongs to the family *Verbenaceae* J. St.-Hil., a large pantropical angiosperm family exhibiting the highest species diversity in tropical and subtropical regions, with only a small number of taxa extending into temperate zones. In China, this family is represented by 175 species, 31 varieties and 10 forms, which are mainly concentrated in the regions south of the Yangtze River [1]. *V. officinalis* is the representative species of this genus. It is widely used in traditional Chinese medicine, especially in ethnic minority areas such as Yunnan, Guangxi and Guizhou, where it is commonly used as a medicinal material. According to traditional Chinese medicine theory, it has a slightly bitter taste and cool medicinal property, with documented meridian tropism towards the liver, spleen and bladder meridians; the definition of meridians derives from the fundamental visceral manifestation theory of traditional Chinese medicine, which divides the human body into interconnected functional pathways for substance transportation and physiological regulation. Its core medicinal indications include clearing heat and detoxifying, relieving sore throat and swelling, promoting blood circulation and removing blood stasis, and promoting urination and reducing damp-heat jaundice, a classical disease definition recorded in ancient Chinese herbal monographs focusing on liver–biliary dampness stagnation-induced icterus [2]. It is commonly used in clinical practice to treat symptoms such as external infection-induced fever, dysentery, malaria, diphtheria, amenorrhea, and abscesses and sores with toxins [2]. In addition, the *V. officinalis* has a neat plant shape, a long-lasting flowering period, and possesses both medicinal and ornamental value. It is also frequently seen in garden landscapes [3].

Modern research has shown that *V. officinalis* contains a rich variety of medicinal substances. The identified components include sesquiterpenoid glycosides, phenylalkanoid glycosides, flavonoids, volatile oils, and triterpenoids, etc. [4,5,6,7,8,9,10,11]. Among them, iridoid glycosides, phenylethylane glycosides and flavonoid components have shown potential for developing treatments for Alzheimer’s disease in improving cognitive function [12]. Flavonoid compounds exhibit significant antibacterial activity against Candida albicans, Escherichia coli and methicillin-resistant Staphylococcus aureus (MRSA) [13]. Monoterpene components are natural anti-cancer active substances [14]. At the same time, in the trace element detection of *V. officinalis*, there are relatively many instances of iron, calcium, magnesium, zinc, manganese, and copper [15,16]. In particular, *V. officinalis* also contains selenium [17], and it has been found to have anti-neoplastic effects in recent years [18], so applying *V. officinalis* to antineoplastic agents has definite research potential.

The systematic identification and precise characterization of chemical components are essential for revealing the pharmacological mechanisms of medicinal plants, developing active ingredients, and establishing quality standards. Ultra-high-performance liquid UHPLC–Q–Exactive–Orbitrap MS features high separation efficiency, high sensitivity and high resolution, and has become a powerful tool for analyzing complex systems of medicinal plants [19]. This technology can obtain the precise molecular weight of compounds through primary mass spectrometry, and make structural inferences by combining the characteristic fragment ions in secondary mass spectrometry. However, the massive raw data, which requires manual screening, is often time-consuming and prone to omissions, thereby limiting the efficiency and accuracy of compound identification. Conventional standalone UHPLC–MS/MS identification depends on successive manual database matching for individual chromatographic peaks; such discrete matching ignores intrinsic structural correlation among analogs and is inefficient when facing abundant isomeric compounds from herbal extracts.

Feature-Based Molecular Networking (FBMN) is a complex sample analysis strategy that has emerged in recent years. Built on the Global Natural Products Social Molecular Network (GNPS) open-access platform, FBMN constructs molecular networks via pairwise MS/MS spectral similarity calculation. Compared with conventional GNPS, FBMN retains chromatographic retention information from UHPLC separation, which enables effective differentiation of isomeric analogs sharing identical precursor *m*/*z* values but distinct chromatographic elution behaviors, thus improving the performance of traditional GNPS for isomer identification to a certain extent [20,21,22,23]. Nevertheless, FBMN still has inherent drawbacks: its clustering accuracy relies heavily on pre-set spectral similarity thresholds and MS/MS fragment matching criteria, and low-abundance metabolites with incomplete fragment spectra are prone to wrong clustering or omission during network construction. Thus, in order to enhance the depth of identification, SIRIUS 6.3.3 (Spectral Inference of Unknowns via Rational Isotope and Fragment Utilization) software was used. It can realize in silico formula and skeleton prediction without relying on physical reference standards through the ZODIAC algorithm (Zero-defect Organic compound Determination via Isotope And Compound clustering). Therefore, this study intends to integrate UHPLC–Q–Exactive–Orbitrap MS, FBMN and SIRIUS software to establish a systematic analytical method: Firstly, high-resolution mass spectrometry data will be obtained through UHPLC–Q–Exactive–Orbitrap MS technology, and preliminary analysis will be conducted in combination with relevant literature. Secondly, a molecular network will be constructed using FBMN to achieve clustering and preliminary screening of compounds. Finally, SIRIUS software will be used to conduct in-depth analysis of each network node, and the structure of unknown compounds will be deduced through molecular formula prediction, isotope pattern matching and fragment ion analysis [24]. This study aims to comprehensively characterize the chemical composition of *V. officinalis*, laying a solid material foundation for subsequent active component screening, pharmacological mechanism research and innovative drug development. It is critical to clarify that total counted compound number from MS detection only reflects detectable metabolite species under current chromatographic and mass spectrometric conditions instead of quantitative concentration of each component inside the plant matrix.

## 2. Results and Discussion

### 2.1. Analytical Strategy

To enhance the depth and accuracy of chemical component identification in *V. officinalis*, we established an integrated analytical strategy based on UHPLC–Q–Exactive–Orbitrap MS coupled with FBMN(GNPS1) and SIRIUS software. First, high-resolution mass spectrometry data were acquired using UHPLC–Q–Exactive–Orbitrap MS, and preliminary annotation was performed by cross-referencing with relevant literature. Subsequently, FBMN was employed to construct a molecular network for compound clustering and preliminary screening. All nodes in the network were classified into two categories strictly following MSI identification standards: MSI Level 2 compounds tentatively identified via published literature MS/MS fragmentation data and GNPS spectral matching, and MSI Level 3 metabolites putatively deduced solely from molecular network clustering correlations combined with SIRIUS in silico prediction. Finally, SIRIUS software was applied for in-depth structural elucidation of each network node, where the structures of unannotated compounds were deduced through integrated analysis of molecular formula prediction, isotope pattern matching and MS/MS fragment ion assignment.

### 2.2. The Chemical Constituent Characterization of V. officinalis

In accordance with MSI identification criteria, a total of 126 chemical constituents were annotated from *V. officinalis*. As summarized in previous phytochemical reviews, flavonoids, terpenoids, phenylpropanoids and phenols dominate the chemical profile of this medicinal herb, and our annotation data are highly consistent with this conclusion.

When comparing the distribution of different compound subclasses, most previous targeted isolation research tended to prioritize readily separable high-content marker compounds such as verbascoside from phenylpropanoids, luteolin and kaempferol from flavonoids, as well as ursolic acid and verbenalin belonging to terpenoid iridoid glycosides. Most early works only obtained fewer than ten isolated structures within phenolic and organic acid subgroups through traditional separation. For phenylpropanoid metabolites, earlier publications mainly documented isolated monomeric caffeic acid derivatives and limited phenylethanoid glycosides including verbascoside and acteoside verified by reference standards; in contrast, many coumarin glycosides and multiple verbascoside stereoisomers annotated in our work could hardly be separated and purified using classic isolation protocols, thus enriching the tentative structural inventory of vervain phenylpropanoids. In the flavonoid subgroup, prior investigations largely focused on easily purified free flavone and flavonol aglycons plus their simple monoglucosides such as apigenin, luteolin and quercetin; supported by FBMN spectral clustering, numerous dimeric flavonoid frameworks and methoxylated flavone positional isomers were putatively deduced in our dataset, extending the known structural diversity of flavonoids from this herb. Terpenoid-related former studies mainly relied on two well-characterized triterpene markers and two common iridoid glycosides obtained via column separation, while a range of triterpene stereoisomers and long-chain unsaturated terpenoid acids observed herein remain rarely described in past phytochemical records and are only structurally inferred from MS/MS evidence. Common simple phenols including gallic acid and salicylic acid were merely noted in preliminary qualitative tests in the previous literature, and our analytical pipeline relying on full MS/MS fragment profiles enabled the tentative differentiation of 18 distinct phenolic monomers and phenolic acid derivatives, most of which lack standard compound validation [25].

Overall, 74 metabolites scarcely recorded in prior phytochemical work were identified in this study, and 15 potential novel compound skeletons were tentatively proposed. The identified compounds were classified into multiple categories: 37 phenylpropanoids, 24 flavonoids, eight organic acids, 24 terpenoids, 18 phenols, five amino acids, one saccharide, and nine other compounds. Phenylpropanoids represented the most abundant annotated class, accounting for 29% of the total identified compounds. Flavonoids, terpenoids, and phenols constituted 19%, 19%, and 14%, respectively, and together formed the core chemical profile of the *V. officinalis* extract. By contrast, organic acids and other components accounted for 6% and 7% respectively, amino acids for 4%, and saccharides only 1%, representing the least abundant category. Collectively, phenylpropanoids, flavonoids, terpenoids, and phenols accounted for 81% of all identified constituents, confirming their status as the primary annotated chemical components of the extract (Figure S1).

**Figure 1:**
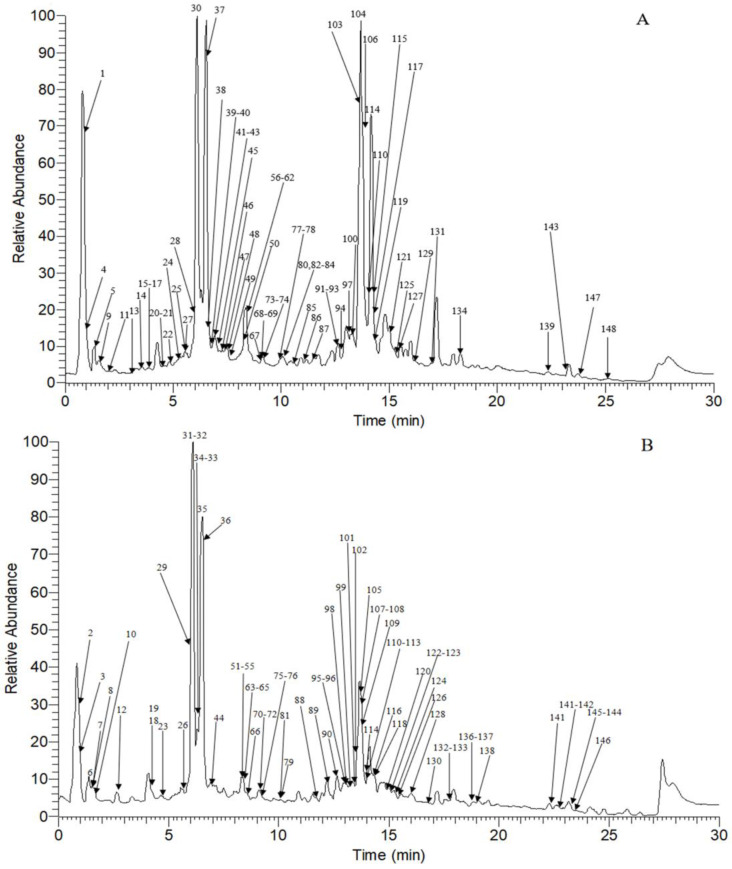
The base peak chromatograms of *V. officinalis* obtained by UHPLC–Q–Exactive–Orbitrap MS. (**A**): negative ion mode; (**B**): positive ion mode.

Among all annotated constituents, 15 were unambiguously characterized as MSI Level 1 by comparison with authentic reference standards, 80 compounds were tentatively identified as MSI Level 2 via consistent literature MS/MS data, chromatographic retention information and GNPS spectral matching, and the remaining 31 metabolites were assigned as putative structures of MSI Level 3 supported solely by SIRIUS in silico prediction without direct experimental confirmation. The chromatographic and mass spectrometric data of the identified components are summarized in Table S1, and the corresponding base peak ion (BPI) chromatogram is presented in Figure 1.

#### 2.2.1. Identification of the Flavonoids in *V. officinalis*

A total of 24 flavonoids were annotated from *V. officinalis*, mainly including simple flavones, flavonols, dihydroflavones and isoflavones. In positive ion mode, [M + H]^+^ is the predominant ion for all four subclasses of flavonoids. Retro-Diels–Alder (RDA) cleavage and C-ring cleavage are the common fragmentation pathways. The flavone nucleus remains relatively stable during fragmentation, while flavonols tend to lose H_2_O first. The saturated C-ring of dihydroflavones makes them more susceptible to ring opening. The unique B-ring position in isoflavones reduces the efficiency of RDA fragmentation, resulting in B-ring rearrangement and direct cleavage becoming the dominant reaction pathways [19,26].

**Figure 2:**
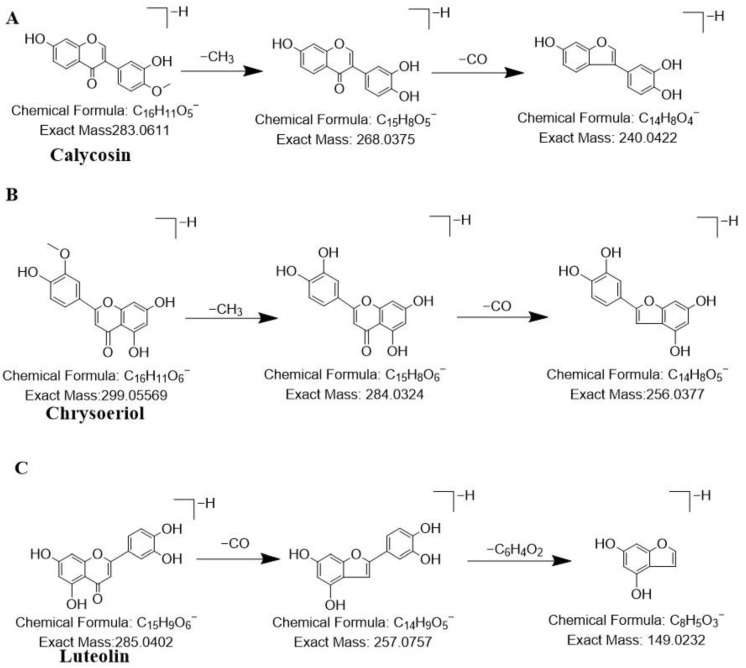
The proposed fragmentation patterns of representative reference compounds. (**A**): Calycosin; (**B**): Chrysoeriol; (**C**): Luteolin.

For compound **100**, a quasi-molecular ion peak at *m*/*z* 283.0611 [M−H]^−^ was detected in the negative ion mode. After losing one methyl group, the ion fragment is *m*/*z* 268.0375, and then after losing one molecule of CO, the ion fragment is *m*/*z* 240.0422. By comparison with the database and reference standards [26], this compound was confirmed as calycosin. Its possible cleavage pathways were shown in Figure 2A. For compound **67**, a quasi-molecular ion peak at *m*/*z* 461.1088 [M−H]^−^ was observed in the negative ion mode. After losing one molecule of glucosyl group, a fragment ion at *m*/*z* 283.0246 was obtained; this fragment further lost one molecule of CO, resulting in a fragment ion at *m*/*z* 255.0293. By comparison with the database, this compound was tentatively characterized chrysoeriol-7-O-*β*-D-glucoside. For compound **92**, a quasi-molecular ion peak at *m*/*z* 331.0800 [M + H]^+^ was detected in the positive ion mode. After losing one molecule of methyl group, a fragment ion at *m*/*z* 316.0565 was obtained; this fragment further lost one molecule of methyl group, resulting in a fragment ion at *m*/*z* 301.0331. By comparison with the database, this compound was tentatively characterized iristetrigenin B.

For compound **87**, a quasi-molecular ion peak at *m*/*z* 299.0556 [M−H]^−^ was found in the negative ion mode. After losing one molecule of methyl group, a fragment ion at *m*/*z* 284.0324 was obtained. Through database and reference standard comparison, this compound was identified as chrysoeriol [27]. Its possible cleavage pathways were shown in Figure 2B. For compound **55**, a quasi-molecular ion peak at *m*/*z* 621.1823 [M−H]^−^ was detected in the negative ion mode. RDA cleavage produced a fragment ion at *m*/*z* 161.0233, and cleavage of the caffeoyl group yielded a fragment ion at *m*/*z* 179.0341. By comparison with the database, this compound was tentatively characterized 3′,4′-dimethoxyluteolin-7-o-neo-hesperidin.

For compound **77**, a quasi-molecular ion peak at *m*/*z* 287.0540 [M + H]^+^ was observed in the positive ion mode. By comparison with the database and reference standard, this compound was identified as kaempferol. For compound **80**, a quasi-molecular ion peak at *m*/*z* 317.0647 [M + H]^+^ was detected in the positive ion mode. In its MS/MS spectrum, the base peak at *m*/*z* 302.0410 was generated by the neutral loss of a methyl group from the methoxy-substituted aromatic ring. Through database and reference standard comparison, this compound was identified as isorhamnetin. For compound **81,** a quasi-molecular ion peak at *m*/*z* 315.0510 [M−H]^−^ was found in the negative ion mode. After losing one molecule of methyl group, a fragment ion at *m*/*z* 300.0273 was obtained. By comparison with the database, this compound was tentatively characterized as eupafolin [28]. For compound **93**, a quasi-molecular ion peak at *m*/*z* 255.0660 [M−H]^−^ was found in the negative ion mode. RDA cleavage produced a B-ring fragment ion at *m*/*z* 135.0075, and further cleavage of the B ring yielded a fragment ion at *m*/*z* 119.0490. By comparison with the database and reference standards, this compound was confirmed as liquiritigenin.

**Figure 3:**
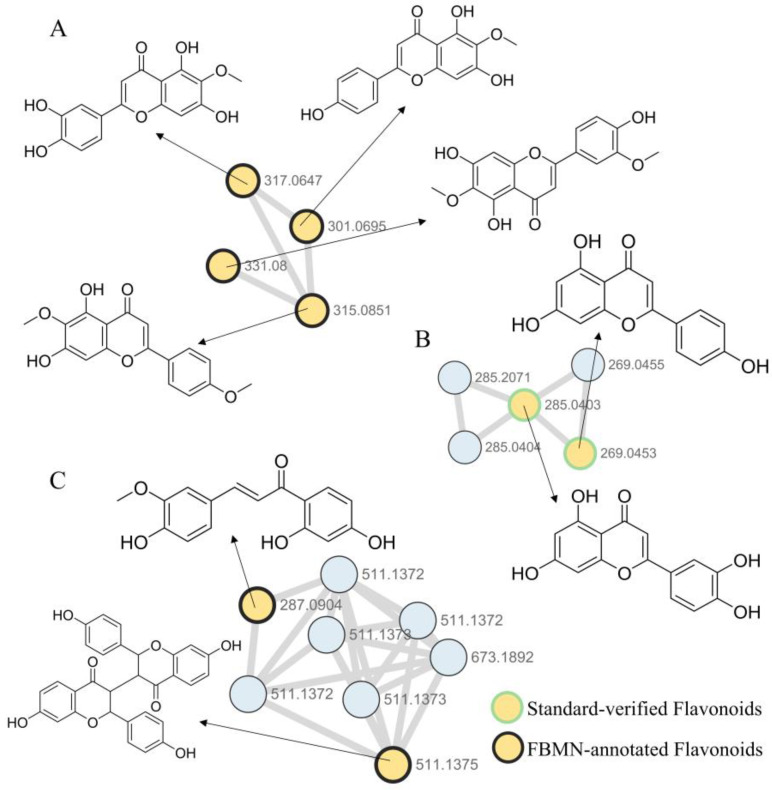
The flavonoid molecular network diagram of *V. officinalis.* (**A**,**C**) Flavonoid molecular network obtained under positive ion mode; (**B**) Flavonoid molecular network obtained under negative ion mode.

On the GNPS platform, Feature-Based Molecular Networking (FBMN) was constructed under both positive and negative ion modes. Through targeted analysis of key core nodes in the molecular networks combined with structural annotation using SIRIUS software, the exact *m*/*z* values and characteristic MS/MS fragment ions of each node were individually matched and validated. Based on FBMN clustering analysis, mass spectrometric data comparison and structural elucidation, flavonoids were found to mainly aggregate in three Molecular clusters. Compounds **79**, **86**, **90**, and **104** were clustered in the same Molecular cluster (Molecular cluster A, Figure 3), with quasi-molecular ion peaks at *m*/*z* 317.0646 [M + H]^+^, *m*/*z* 301.0695 [M + H]^+^, *m*/*z* 331.0800 [M + H]^+^, and *m*/*z* 315.0851 [M + H]^+^, respectively. Compounds **78**, **98**, **84** and **96** were clustered in the same molecular cluster (Molecular cluster B, Figure 3). In negative ion mode, their quasi-molecular ion peaks were *m*/*z* 285.0403 [M−H]^−^, *m*/*z* 285.0403 [M−H]^−^, *m*/*z* 269.0453 [M−H]^−^, and *m*/*z* 269.0453 [M−H]^−^, respectively. Among them, compounds **78** and **98** had identical molecular formulas, highly similar MS/MS fragmentation patterns and different chromatographic retention times under the optimized UHPLC conditions, suggesting they were hydroxyl positional isomers; compounds **84** and **96** had identical molecular formulas, homologous fragmentation characteristics and distinct elution profiles, confirming they were flavone–isoflavone skeleton isomers. All compounds in these two clusters shared a benzopyranone parent nucleus substituted with multiple hydroxyl and methoxy groups. The conjugated system formed by the benzene and pyranone rings stabilized the fragment ions, with no random carbon chain cleavage observed. All fragments retained the aromatic or heterocyclic core structure, and fragmentation mainly involved neutral loss of methoxy and hydroxyl groups, followed by inter-ring bond cleavage of the parent nucleus. For example, in negative ion mode, the quasi-molecular ion peak of compound **78** was *m*/*z* 285.0402 [M−H]^−^. Firstly, the luteolin parent nucleus underwent RDA cleavage with neutral loss of CO, forming the deprotonated fragment ion at *m*/*z* 257.0757. Subsequently, the B-ring moiety was eliminated via neutral loss of the C_6_H_4_O_2_ fragment, generating the diagnostic A-ring derived fragment at *m*/*z* 149.023. Finally, further neutral loss of an oxygen atom occurred to produce the stable aromatic fragment ion at *m*/*z* 133.0283. This compound was unambiguously confirmed as luteolin by comparison with database spectra and authentic reference standards, and its proposed fragmentation pathways are shown in Figure 2C [27]. Based on SIRIUS prediction and database matching, the remaining compounds were tentatively annotated as follows: **79**: nepetin; **86**: hispidulin; **90**: jaceosidin; **104**: 5,7-dihydroxy-6-methoxy-2-(4-methoxyphenyl)chromen-4-one; **98**: 2-(2,5-dihydroxyphenyl)-5,7-dihydroxy-4H-1-benzopyran-4-one; **84**: apigenin [29]; **96**: genistein.

Compounds **83**, **89**, **97**, **99**, **101**, **106** and **95** were clustered in the same Molecular cluster (Molecular cluster C, Figure 3). In positive ion mode, their quasi-molecular ion peaks were *m*/*z* 511.1376 [M + H]^+^, *m*/*z* 511.1372 [M + H]^+^, *m*/*z* 511.1373 [M + H]^+^, *m*/*z* 511.1372 [M + H]^+^, *m*/*z* 511.1373 [M + H]^+^, *m*/*z* 511.1371 [M + H]^+^, *m*/*z* 287.0904 [M + H]^+^. Among them, compounds **83**, **89**, **97**, **99**, **101** and **106** had identical molecular formulas, highly homologous MS/MS fragmentation patterns and distinct chromatographic retention behaviors, indicating they constituted a mixed isomer group of lophirone-type flavonoid dimers, including skeleton isomerism, positional isomerism and geometric isomerism. All compounds in this cluster relied on conjugated systems to stabilize fragment ions, with no random carbon chain cleavage. All fragments retained the core ring system structure, and cleavage occurred preferentially at side chain C-C bonds or functional group linkage bonds, presenting a stepwise cleavage pattern. For example, in compound **83**, the base peak at *m*/*z* 137.0229 in its MS/MS spectrum was derived from cleavage of the central furan/benzofuran bridge and dissociation of the two aromatic monomer units. The fragment ion at *m*/*z* 255.0645 was formed by symmetric cleavage of the dimer skeleton, while *m*/*z* 155.0336 was attributed to further loss of hydroxyl and carbonyl groups from the aromatic ring. Through SIRIUS matching and database comparison, this compound was tentatively identified as 7-hydroxy-3-[7-hydroxy-2-(4-hydroxyphenyl)-4-oxo-3,4-dihydro-2H-1-benzopyran-3-yl]-2-(4-hydroxyphenyl)-3,4-dihydro-2H-1-benzopyran-4-one. Based on SIRIUS prediction and database matching, the remaining compounds were tentatively annotated as follows: **89**: (2S,3S)-3-(2,4-dihydroxybenzoyl)-2,7-bis(4-hydroxyphenyl)-2,3,6,7-tetrahydrofuro [3,2-g]chromen-5-one; **97**: 1-[3-(2,4-dihydroxybenzoyl)-6-hydroxy-2-(4-hydroxyphenyl)-2,3-dihydro-1-benzofuran-5-yl]-3-(4-hydroxyphenyl)prop-2-en-1-one; **99**: (2S,3R)-3-[5-[(E)-3-(2,4-dihydroxyphenyl)-3-oxoprop-1-enyl]-2-hydroxyphenyl]-7-hydroxy-2-(4-hydroxyphenyl)-2,3-dihydrochromen-4-one; **101**: (E)-1-[3-(2,4-dihydroxybenzoyl)-4-hydroxy-2-(4-hydroxyphenyl)-2,3-dihydro-1-benzofuran-5-yl]-3-(4-hydroxyphenyl)prop-2-en-1-one; **106**: 3-[3-(2,4-dihydroxybenzoyl)-2-(4-hydroxyphenyl)-2,3-dihydro-1-benzofuran-5-yl]-1-(2,4-dihydroxyphenyl)prop-2-en-1-one; **95**: homobutein.

#### 2.2.2. Identification of the Phenylpropanoids in *V. officinalis*

A total of 37 phenylpropanoid compounds were identified from the *V. officinalis*, mainly including simple phenylpropanoids, lignans and coumarins. Simple phenylpropanoids have a C_6_-C_3_ monomer as the skeleton and tend to undergo benzyl cleavage and lose neutral fragments such as CO_2_ in the negative ion mode. Lignans are C_6_-C_3_ unit polymers, and in the positive ion mode, the main fragmentation occurs at the β-O-4 connection bond, generating characteristic phenylpropanoid unit ions. Coumarins have a benzopyranone structure, and in the positive ion mode, the typical fragmentation pathway is the consecutive loss of two molecules of CO. It can be combined with the fragment ions and substituent loss of RDA cleavage to achieve rapid identification.

For compound 46, a quasi-molecular ion peak at *m*/*z* 181.0495 [M + H]^+^ was observed in positive ion mode. The precursor ion first lost one molecule of H_2_O to generate the fragment ion at *m*/*z* 163.0384, which underwent further loss of one H_2_O molecule to produce the fragment ion at *m*/*z* 145.0280. In parallel, the fragment ion at *m*/*z* 163.0384 underwent successive eliminations of CO molecules, yielding the fragment ion at m/z 135.0436 after the first CO loss and the fragment ion at *m*/*z* 107.0491 after the second CO loss. By comparison with the database, this compound was tentatively characterized as caffeic acid or its isomer. For compound **33**, a quasi-molecular ion peak at *m*/*z* 195.0645 [M + H]^+^ was observed in the positive ion mode. After losing one molecule of H_2_O, a fragment ion at *m*/*z* 177.0541 was obtained; this fragment further lost one molecule of CO, resulting in a fragment ion at *m*/*z* 149.0592. By comparison with the database, this compound was tentatively characterized as ferulic acid [30].

**Figure 4:**
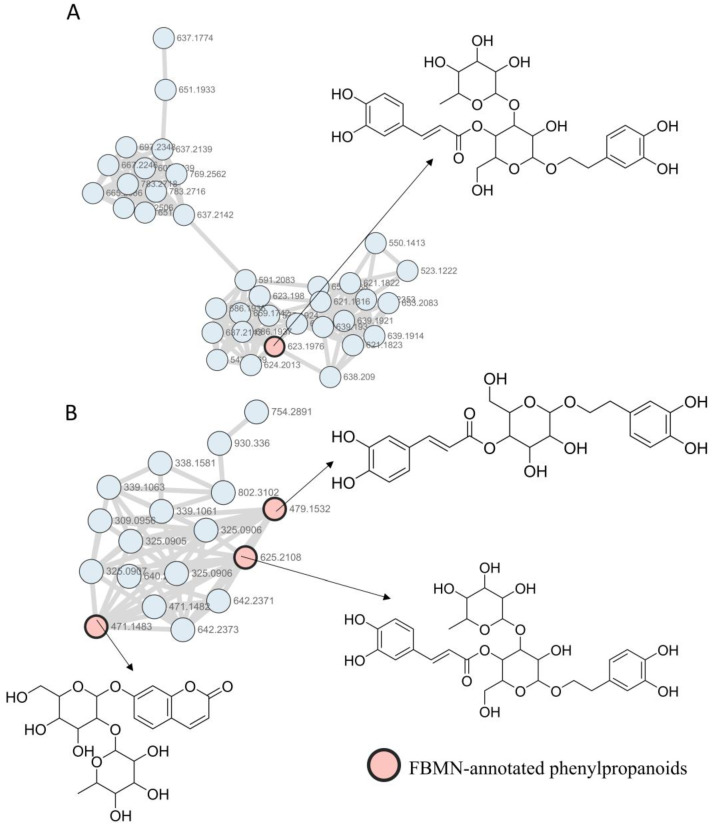
The phenylpropanoids molecular network diagram of *V. officinalis.* (**A**) Phenylpropanoid molecular network obtained under negative ion mode; (**B**) Phenylpropanoid molecular network obtained under positive ion mode.

Based on FBMN molecular network clustering analysis, mass spectrometric data comparison and structural elucidation, phenylpropanoids were found to mainly aggregate in two Molecular clusters. Compounds **64**, **72**, **39**, **47**, **57**, **71**, **48**, **56**, **50**, **58**, **49** and **52** were clustered in the same molecular cluster (Molecular cluster B, Figure 4). In positive ion mode, their quasi-molecular ion peaks were *m*/*z* 339.1062 [M + H]^+^, *m*/*z* 339.1062 [M + H]^+^, *m*/*z* 325.0906 [M + H]^+^, *m*/*z* 325.0904 [M + H]^+^, *m*/*z* 325.0905 [M + H]^+^, *m*/*z* 325.0906 [M + H]^+^, *m*/*z* 471.1481 [M + H]^+^, *m*/*z* 471.1481 [M + H]^+^, *m*/*z* 625.2106 [M + H]^+^, *m*/*z* 625.2104 [M + H]^+^, *m*/*z* 625.2106 [M + H]^+^, and *m*/*z* 479.1532 [M + H]^+^, respectively. Among them, compounds **64** and **72** had highly similar MS/MS fragmentation patterns and distinct chromatographic retention times, indicating that they were positional isomers; compounds **39**, **47**, **57** and **71** shared homologous fragmentation characteristics and different elution sequences, and were thus identified as skeleton isomers; compounds **48** and **56,** as well as compounds **50** and **58,** also showed consistent cleavage patterns and varied retention behaviors, and were recognized as two pairs of glycosidic isomers. Compounds **69** and **74** were clustered in Molecular cluster A (Figure 4). Both exhibited a quasi-molecular ion peak at *m*/*z* 623.1978 [M−H]^−^ in negative ion mode, and their similar secondary fragmentation characteristics combined with significant retention differences confirmed they were stereoisomers. All compounds in these two clusters followed a consistent fragmentation rule: preferential cleavage of side chains or glycosidic bonds occurred first, followed by parent nucleus ring-opening, and finally neutral loss of hydroxyl, carbonyl and other functional groups. All glucose- or rutinose-containing compounds generated characteristic sugar-derived fragment ions at *m*/*z* 85, 71 and 113, and the sugar moiety typically dissociated directly upon glycosidic bond cleavage without participating in subsequent fragmentation of the aglycone. For example, in compound **64**, the ion fragment at *m*/*z* 145.0279 was produced after cleavage of the C-C bond in the cyclohexane ring; *m*/*z* 177.0540 was the fragment ion formed by loss of the acryloyloxy group; *m*/*z* 117.0333 and *m*/*z* 97.0285 were the products of further dehydroxylation and dehydration of the cyclohexane ring. Through SIRIUS matching and database comparison, this compound was determined as (1R,3R,4R,5R)-3-[3-(3,4-dihydroxyphenyl)prop-2-enoyloxy]-4,5-dihydroxycyclohexane-1-carboxylic acid. Based on SIRIUS prediction and database matching, the remaining compounds were tentatively annotated as follows: **72**: 3-p-coumaroylquinic acid; **39**: 8-[3,4,5-trihydroxy-6-(hydroxymethyl)oxan-2-yl]oxychromen-2-one; **47**: 3-hydroxycoumarin glucoside; **57**: mahaleboside; **71**: 7-[3,4,5-trihydroxy-6-(hydroxymethyl)oxan-2-yl]oxychromen-2-one; **48**: ((2R,3R,4S,5R,6R)-6-(2-(3,4-dihydroxyphenyl)ethoxy)-3,5-dihydroxy-4-((2R,3R,4R,5R,6S)-3,4,5-trihydroxy-6-methyloxan-2-yl)oxyoxan-2-yl)methyl (E)-3-(3,4-dihydroxyphenyl)prop-2-enoate; 56: umbelliferone 7-O-rutinoside; **50**, **49**, **69** and **58**: verbascoside isomers (reclassified after retention time cross-check with verbascoside reference standard); **52**: calceolarioside A; **74**: isoacteoside.

#### 2.2.3. Identification of the Phenols in *V. officinalis*

A total of 18 phenols compounds were identified in the *V. officinalis*, mainly consisting of monophenols and polyphenols. Among them, phenolic acids accounted for a relatively large proportion. The phenolic acid compounds in the structure mostly contain phenolic hydroxyl groups and carboxyl groups, and are prone to lose CO_2_, H_2_O, CO, HCOOH and other groups during decomposition [31].

**Figure 5:**
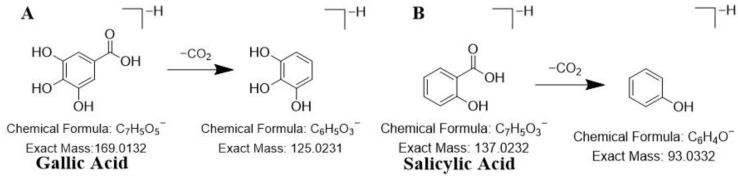
The proposed fragmentation patterns of representative reference compounds. (**A**): gallic acid; (**B**): salicylic acid.

For compound **11**, a quasi-molecular ion peak at *m*/*z* 169.0132 [M−H]^−^ was observed in the negative ion mode. After losing one molecule of CO_2_, a fragment ion at *m*/*z* 125.02316 was obtained; this fragment ion further lost one molecule of H_2_O, resulting in a fragment ion at *m*/*z* 107.5724. By comparison with the database and reference standards, this compound was confirmed as gallic acid. Its possible cleavage pathways were shown in Figure 5A. For compound **61**, a quasi-molecular ion peak at *m*/*z* 137.0232 [M−H]^−^ was detected in the negative ion mode. After losing one molecule of CO_2_, a fragment ion at *m*/*z* 93.0332 was obtained. By comparing with the database and reference standards, this compound was identified as salicylic acid [32]. Its possible cleavage pathways were shown in Figure 5B.

**Figure 6:**
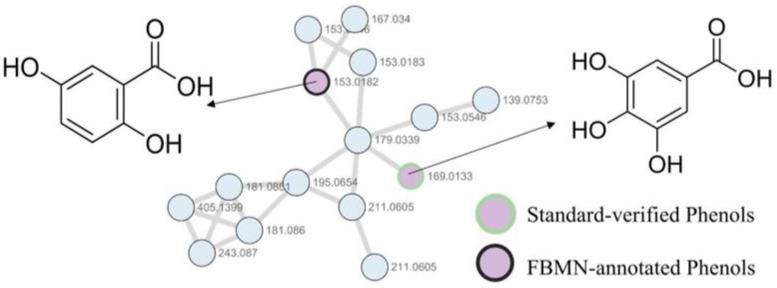
The phenols molecular network diagram of *V. officinalis* in negative ion mode.

Based on FBMN molecular network clustering analysis, mass spectrometric data comparison and structural elucidation, phenolic compounds were found to mainly aggregate in one Molecular cluster. Compounds **22** and **11** were clustered in the same Molecular cluster (Molecular cluster A, Figure 6). In negative ion mode, their quasi-molecular ion peaks were *m*/*z* 153.0182 [M−H]^−^ and *m*/*z* 169.0132 [M−H]^−^, respectively. The fragmentation of these two compounds mainly occurred at functional group linkage bonds, with neutral loss of CO, CO_2_, H_2_O and methoxy groups as the dominant pathways. Compound **22** was characterized by the generation of a fragment ion at *m*/*z* 108.0203 via side chain cleavage. Through SIRIUS software matching and database comparison, these two compounds were tentatively annotated as gallic acid and gentisic acid or its isomer.

#### 2.2.4. Identification of the Terpenoids in *V. officinalis*

A total of 24 terpenoids were identified from the *V. officinalis*, mainly including iridoids and triterpenoids. Terpenoids are prone to consecutive dehydration, demethylation, ring-opening cleavage and side chain cleavage. Iridoids, as derivatives of cyclopentane monoterpene, mostly exist in the form of glycosides and preferentially undergo glycosidic bond cleavage to lose sugar groups, accompanied by characteristic cleavage such as ring opening, decarboxylation and dehydration of the parent nucleus. Triterpenoids, with their multi-ring rigid skeletons, mainly undergo consecutive dehydration, methylation loss, ring cleavage and skeleton rearrangement to generate a series of highly stable terpenoid skeleton characteristic ions [33].

For compound **109**, a quasi-molecular ion peak at *m*/*z* 469.3319 [M−H]^−^ was found in the negative ion mode. By comparison with the database and reference standards, this compound was identified as glycyrrhetinic acid. For compound **121**, a quasi-molecular ion peak at *m*/*z* 455.3526 [M−H]^−^ was detected in the negative ion mode. Through database and reference standard comparison, this compound was identified as ursolic acid. For compound **120**, a quasi-molecular ion peak at *m*/*z* 455.3503 [M−H]^−^ was observed in the negative ion mode. By comparison with the database and reference standards, this compound was confirmed as betulinic acid. For compound **111**, a quasi-molecular ion peak at *m*/*z* 453.3369 [M + H]^+^ was found in the positive ion mode. Dehydration or decarboxylation of the aglycone produced a fragment ion at *m*/*z* 135.1164; epoxy ring opening or side chain loss yielded a fragment ion at *m*/*z* 135.1164. By comparison with the database, this compound was tentatively characterized wilforlide A. For compound **24**, a quasi-molecular ion peak at *m*/*z* 153.1269 [M + H]^+^ was found in the positive ion mode. After losing one molecule of aldehyde group, a fragment ion at *m*/*z* 109.1012 was obtained. By comparison with the database, this compound was tentatively characterized as citral [19].

**Figure 7:**
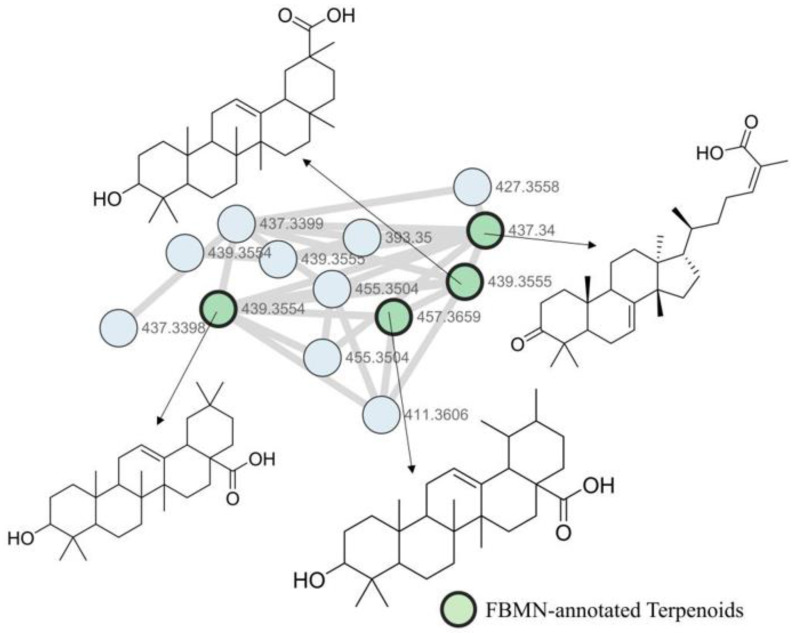
The terpenoids molecular network diagram of *V. officinalis* in positive ion mode.

Based on the FBMN molecular network clustering analysis, mass spectrometry information comparison and structure derivation, it was annotated that terpene compounds mainly aggregated in one Molecular cluster. Compounds **118**, **119**, **122**, **124**, **110**, **113**, **123** and **114** were aggregated in the same Molecular cluster (Molecular cluster A, Figure 7). Under positive ion mode, their quasi-molecular ion peaks were *m*/*z* 439.3555 [M + H]^+^, *m*/*z* 439.3555 [M + H]^+^, *m*/*z* 439.3555 [M + H]^+^, *m*/*z* 439.3555 [M + H]^+^, *m*/*z* 455.3504 [M + H]^+^, *m*/*z* 455.3503 [M + H]^+^, *m*/*z* 457.3660 [M + H]^+^, and *m*/*z* 437.3399 [M + H]^+^, respectively. Among them, compounds **118**, **119**, **122** and **124** had similar secondary fragment characteristics and varied retention behaviors, suggesting they are four triterpenoid stereoisomers; compounds **110** and **113** had similar secondary fragment characteristics and different elution time, confirming as positional isomers. All the compounds in this Molecular cluster were triterpenoids, with a core structure of a densely ringed triterpene nucleus containing functional groups such as ketone and hydroxyl groups. The fragmentation rules revolved around the preferential cleavage of side chains of the nucleus, selective heterolytic cleavage of inter-ring bonds, and ordered loss of functional groups. For example, in compound **114**, the side chain was preferentially cleaved, generating the *m*/*z* 95.0857 ion fragment. *m*/*z* 107.0855 and *m*/*z* 119.0854 were local fragments of the nucleus. Through SIRIUS matching and database comparison, it was determined that this compound was (Z,6S)-2-methyl-6-[(10R,13S,14S,17S)-4,4,10,13,14-pentamethyl-3-oxo-1,2,5,6,9,11,12,15,16,17-decahydrocyclopenta[a]phenanthren-17-yl]hept-2-enoic acid. Based on SIRIUS prediction and database matching, the remaining compounds were tentatively annotated as follows: **118**: 18-hydroxy-1,2,5,8,15,19,19-heptamethylpentacyclo [12.8.0.0<2,11>.0<5,10>.0<15, 20>]docos-11-ene-8-carboxylic acid; **119**: 2-(3a,5a,5b,8,8,11a-hexamethyl-9-oxo-2,3,4,5,6,7,7a,10,11,11b,12,13,13a,13b-tetradecahydro-1H-cyclopenta[a]chrysen-1-yl)prop-2-enal; **122**: 3-Hydroxyolean-12-en-28-oic acid; **124**: betulonal; **110**: ursonic acid; **113**: (+)-betulonic acid; **123**: (1S,2R,4aR,6aR,6aS,6bR,8aR,10R,12aR,14bR)-10-hydroxy-1,2,6a,6b,9,9,12a-heptamethyl-2,3,4,5,6,6a,7,8,8a,10,11,12,13,14b-tetradecahydro-1H-picene-4a-carboxylic acid.

#### 2.2.5. Identification of the Organic Acids in *V. officinalis*

**Figure 8:**
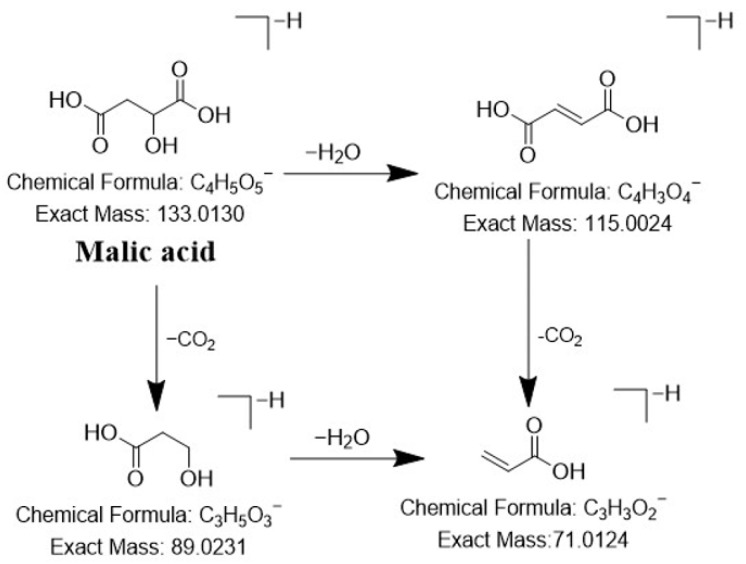
The proposed fragmentation patterns of malic acid.

For compound **4**, a quasi-molecular ion peak at *m*/*z* 133.0130 [M−H]^−^ was observed in the negative ion mode. After losing one molecule of H_2_O, a fragment ion at *m*/*z* 115.0024 was obtained. And then losing one molecule of CO_2_, a fragment ion at *m*/*z* 71.0124 was obtained. By comparing with the database and reference standards, this compound was identified as malic acid. Its possible cleavage pathways were shown in Figure 8. For compound **70**, a quasi-molecular ion peak at *m*/*z* 151.1112 [M + H]^+^ was observed in the positive ion mode. The fragment ion at *m*/*z* 133.1006 was generated by neutral loss of one H_2_O molecule from the [M + H]^+^ precursor ion. Subsequent loss of one C_2_H_2_ molecule from the *m*/*z* 133.1006 ion yielded the fragment ion at *m*/*z* 107.0854, which further eliminated one CO molecule to produce the fragment ion at *m*/*z* 79.0546. By comparison with the database, this compound was tentatively characterized as perillene.

**Figure 9:**
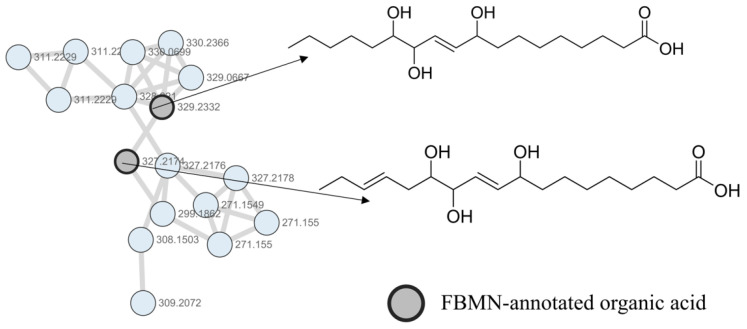
The organic acids molecular network diagram of *V. officinalis* in negative ion mode.

The organic acids identified in the *V. officinalis* through FBMN are mainly concentrated in one Molecular cluster, compounds **91** and **85** as shown in the Molecular cluster A in Figure 9. In the negative ion mode, their quasi-molecular ion peaks are *m*/*z* 329.2332 [M−H]^−^ and *m*/*z* 327.2174 [M−H]^−^, respectively. The fragmentation patterns of both compounds follow the rule of preferentially breaking the carbon chain at the position substituted by hydroxyl or double bond, followed by stepwise carbon chain cleavage and then dehydration or dehydroxylation of hydroxyl groups. Both generate *m*/*z* 211.1332 and *m*/*z* 171.1016 hydroxyalkyl fragments, which are characteristic ions of the category of trihydroxy octadecanoic acids. Through SIRIUS matching and database comparison, it can be determined that the compounds are 9,12,13-Trihydroxyoctadec-10-enoic acid and 9,12,13-Trihydroxy-10,15-octadecadienoic acid, respectively.

## 3. Materials and Methods

### 3.1. Chemicals, Reference Standard and Materials

The HPLC-grade acetonitrile and methanol were purchased from Mackin company. The MS-grade formic acid was obtained from the Thermo Fisher Scientific Co., Ltd. (San Jose, CA, USA). The deionized water was purchased from Watsons Water (Guangzhou, China). Other solvents were of an analytical grade. *V. officinalis*, including root, stem, leaf and flower, were collected from Xupu County, Huaihua City, Hunan Province. The samples were acquired in May 2023 with the local ecological and cultural standards under the supervision of Prof. Wei Cai, Pharmacognosist and at the School of Pharmaceutical Sciences of the Hunan University of Medicine, Hunan, China. The plant specimen of *V. officinalis* is stored in the Pharmacognosy Specimen Room of the School of Pharmaceutical Sciences of the Hunan University of Medicine. The collection of plant materials was collected following international, national, and local legislation. Details of the 15 reference standards are provided in Table S2.

### 3.2. Standard and Sample Preparation

*V. officinalis* was pulverized into fine powder and extracted via ultrasonic-assisted extraction with 70% aqueous methanol at a solid–liquid ratio of 1:10 (g/mL) at room temperature for 30 min each time. Three parallel samples were prepared and all supernatants from three extractions were combined, collected and filtered to prepare the test solution for subsequent analysis.

### 3.3. Instruments and LC–MS/MS Conditions

An Dionex Ultimate 3000 UPLC (Thermo Fisher Scientific, San Jose, CA, USA) and the Q-Exactive Focus Orbitrap MS (Thermo Fisher Scientific, San Jose, CA, USA), equipped with an electrospray ionization (ESI) source, were used to acquire the MS and MS^2^ data of *V. officinalis*.

An Thermo Scientific Hypersil GOLD™ aQ (100 mm × 2.1 mm, 1.9 μm) was applied for chromatographic separation with a column temperature of 40 °C with a flow rate of 0.3 mL/min. The mobile phase consisted of water containing 0.1% formic acid as eluent A and acetonitrile as eluent B. The flow rate was set at a linear gradient as follows: 95–90% A at 0–2 min; 90–80% A at 2–5 min; 80–75% A at 5–10 min; 75–45% A at 10–12 min; 45–20% A at 12–20 min; 20–5% A at 20–25 min; 5–95% A at 25–26 min; 26–30 min, phase A was maintained at 95%. The sample injection volume was 2 μL.

MS analysis in both positive and negative ionization modes using an electrospray ionization (ESI). The key parameters were as follows: spray voltage, 3.5 kV (+); spray voltage, 3.2 kV (−); the sheath gas flow rate, 35 arb; aux gas flow rate, 10 arb; capillary temperature, 320 °C; heater temperature, 350 °C; S-lens RF level, 60. The MS spectra were recorded in Full MS-ddMS^2^ mode; the scan range was *m*/*z* 100–1500. The stepped, normalized collision energies were 20%, 40%, and 60%. Data acquisition and processing were carried out with the Xcalibur version 4.2 (Thermo Fisher Scientific, San Jose, CA, USA).

### 3.4. Data Processing and Analysis

MS data processing and compound annotation were performed using the open-source MZmine 4.8.0 framework. LC-MS/MS raw data acquired on a Dionex Ultimate 3000 UPLC system coupled to a Q-Exactive Focus Orbitrap mass spectrometer were directly imported into MZmine in Thermo.raw format with vendor centroiding enabled.

Positive- and negative-ion datasets were processed separately throughout the workflow. Mass detection was performed using the Auto mass detector, with noise levels set to 1.0 × 10^5^ for MS1 scans and 100 for MS2 scans. Chromatograms were constructed from MS1 data using a minimum of 8 consecutive scans, a minimum intensity of 3.0 × 10^3^, a minimum absolute height of 7.0 × 10^3^, and a scan-to-scan *m*/*z* tolerance of 0.002 Da or 10 ppm.

Chromatographic peak deconvolution was carried out using the local minimum feature resolver. The chromatographic threshold was set to 90%, the minimum retention time search range was set to 0.05 min, the peak duration range was set to 0.00–1.00 min, and a minimum of 8 data points were required for peak detection. MS/MS scan pairing was enabled with an MS1-to-MS2 precursor tolerance of 0.01 Da or 10 ppm.

Isotopic features were processed using the ^13^C isotope filter with an *m*/*z* tolerance of 0.001 Da or 3.5 ppm and a retention time tolerance of 0.01 min. Feature alignment was then performed using the join aligner with an *m*/*z* tolerance of 0.001 Da or 5 ppm and a retention time tolerance of 0.10 min. The aligned feature lists were further filtered by retaining features within the retention time range of 0.50–27.00 min and requiring the presence of MS/MS spectra. The processed positive- and negative-ion feature lists were exported separately as MGF files for subsequent MS/MS-based annotation and molecular networking analysis.

For FBMN (GNPS available online: https://bio.tools/gnps, accessed on 12 January 2026) analysis, the precursor ion and fragment ion mass tolerances were both set to 0.02 Da. Molecular network edges were retained when the cosine score was greater than 0.7 and more than 6 fragment ions were matched. The resulting networks were downloaded from the job page and imported into Cytoscape 3.9.1 for visualization and further analysis. Compounds were putatively annotated based on their MS/MS fragmentation patterns, molecular ion information, retention time, and previously reported literature. All metabolites predicted only by software without reference standard validation were marked as tentatively or putatively annotated rather than unambiguously identified.

MGF files were imported into SIRIUS 6.3.3 software and the “Compute All” workflow was executed. The global configuration was set as follows: instrument type was Orbitrap; MS2 mass accuracy was 5 ppm; fallback adducts were [M−H]^−^ and [M + H]^+^; all databases were selected; spectral matching was enabled with a precursor deviation of 10 ppm. For the molecular formula identification module: SIRIUS and ZODIAC algorithms were enabled; the strategy was set to “De novo + bottom-up (recommended)”; “Perform de novo below *m*/*z*” was set to 1000 and the element filter was applied; allowed elements included H, C, N, O (automatic detection was disabled). Additionally, the Predict, Score threshold, Search:DBs and MSNovelist modules were enabled, and the “PubChem as fallback” option was disabled. It should be noted that structural predictions from SIRIUS and ZODIAC cannot replace physical confirmation via reference standards or nuclear magnetic resonance (NMR) experiments; all unvalidated annotated structures correspond to putative MSI Level 3 assignments.

## 4. Conclusions

This study established an integrated analytical strategy of UHPLC–Q–Exactive–Orbitrap MS-FBMN-SIRIUS, which systematically annotated 126 compounds in the 70% aqueous methanol extract of *V. officinalis*. These compounds covered more than 10 major categories such as phenylpropanoids, flavonoids, terpenoids and phenols, providing the core chemical basis for the pharmacological effects underlying the traditional clinical application of *V. officinalis*. By leveraging the chromatographic retention data retained in FBMN, this strategy broke through the inherent limitations of traditional molecular networking in isomer discrimination, significantly improved the efficiency and accuracy of component identification in complex traditional Chinese medicine systems, and elucidated the characteristic fragmentation rules of major component classes. It provides a referable technical paradigm and theoretical basis for the rapid qualitative analysis of similar natural products.

Nevertheless, this integrated workflow still has non-negligible limitations. On the one hand, for structurally complex polycyclic natural products, SIRIUS-based computer-aided structure prediction is prone to ambiguous skeleton assignment in the absence of corroboration from NMR or authentic reference standard data. On the other hand, the clustering accuracy of FBMN is restricted by preset spectral matching thresholds, leading to occasional misclustering of low-abundance structural analogs.

This study systematically enriches the chemical constituent database of *V. officinalis*, laying a solid foundation for its pharmacodynamic substance screening, establishment of quality standard systems and innovative drug development.

## Data Availability

The original contributions presented in this study are included in the article/Supplementary Materials. Further inquiries can be directed to the corresponding authors.

## Supplementary Materials

The following supporting information can be downloaded at: https://www.mdpi.com/article/10.3390/molecules31132244/s1, Figure S1: Proportion of different chemical constituents of *V. officinalis*; Table S1: Compounds identified by UHPLC-Q-Exactive Orbitrap MS from whole plant of *V. officinalis*; Table S2: Detailed information of the 15 reference standards; Table S3. Abbreviation list. References [34,35,36,37,38,39,40,41,42,43,44,45,46,47] are cited in the Supplementary Materials.
