# Supplementary material for: Systematic Identification of Chemical Components and Analysis of Major Constituents of Verbena officinalis L. Based on UHPLC–Q–Exactive–Orbitrap MS Combined with Feature-Based Molecular Networking and SIRIUS Strategy
Source: Molecules. 2026 Jun 25;31(13):2244. doi: 10.3390/molecules31132244 (PMC13363405; doi:10.3390/molecules31132244)
Supplement: Supplementary file 1 [file molecules-31-02244-s001.zip › molecules-4336549-supplementary.pdf]

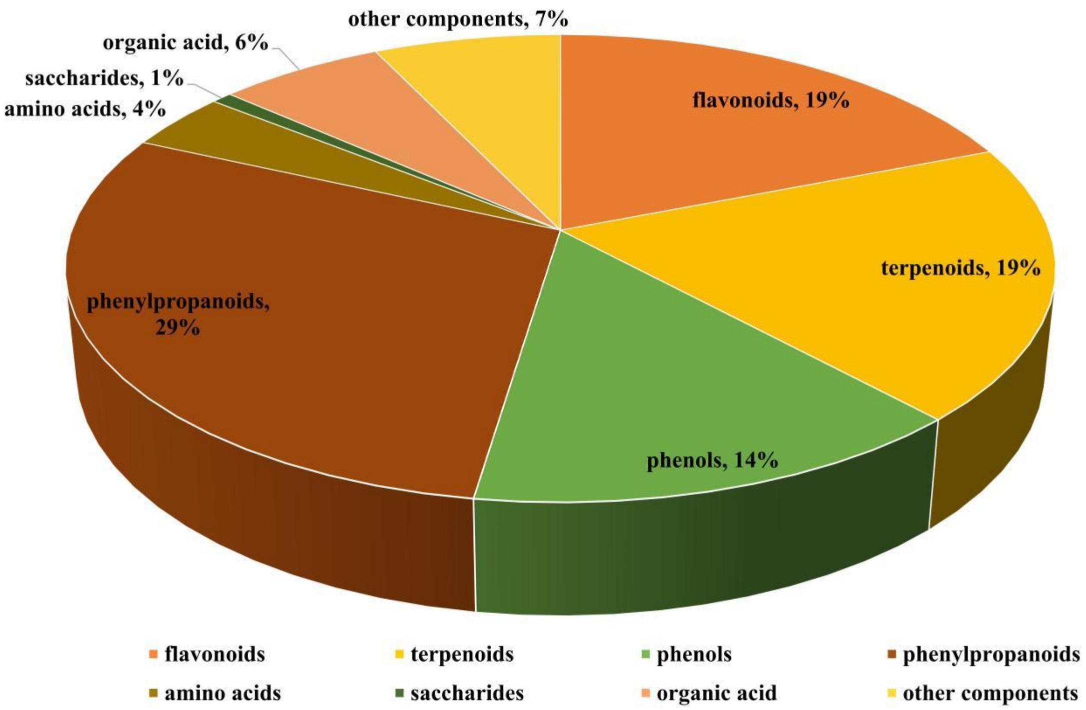

Figure S1. Proportion of different chemical constituents of *V. officinalis* (Note: This statistical pie chart only displays detected compound species proportion rather than relative quantitative abundance of each constituent class)

Table S1. Compounds identified by UHPLC-Q-Exactive Orbitrap MS from whole plant of V. officinalis

Peak	tR	Teoretical mass (m/z)	Experimental mass (m/z)	Error (ppm)	Formula	MS/MS fragment (-)	MS/MS fragment (+)	Identifcation	References	Compounds class
1	0.90	341.1089	341.1079	0.212	$C_{12}H_{22}O_{11}$	MS^2[341]:59.0124(100),101.0231(15),113.0231(12),119.0341(5)		Trehalose	[1]	Saccharides
2	0.91	116.0706	116.0705	-0.475	$C_5H_9NO_2$		MS^2[116]:70.0656(100),116.0705(18),	Proline	[2]	Amino acids
3	0.96	136.0617	136.0614	-2.144	$C_5H_5N_5$		MS^2[136]:136.0614(100)	Adenine	[3]	Other
4*	0.97	133.0142	133.0130	-0.900	$C_4H_6O_5$	MS^2[133]:115.0024(100),133.0132(15),72.9917(18),71.0124(95),89.0231(8)		Malic acid	[2]	organic acid
5	1.39	243.0622	243.0619	3.116	$C_9H_{12}N_2O_6$	MS^2[243]:82.0284(54),110.0234(100),122.0235(15),140.0347(3),152.0345(5)		Uridine	[2]	Other
6	1.40	182.0811	182.0807	-2.470	$C_9H_{11}NO_3$		MS^2[182]:136.0753(100),119.0490(61),123.0438(69),107.0491(1)	Tyrosine	[3]	Amino acids
7	1.49	268.1040	268.1033	-2.724	$C_{10}H_{13}N_5O_4$		MS^2[268]:136.0614(100),119.0351(1),	Adenosine	[3]	Other
8	1.52	132.1019	132.1016	-2.235	$C_6H_{13}NO_2$		MS^2[132]:86.0967(100),	Leucine	[2]	Amino acids
9	1.63	282.0843	282.0842	3.421	$C_{10}H_{13}N_5O_5$	MS^2[282]:150.0410(100),133.0144(48)		Guanosine	[4]	Other
10	1.64	152.0566	152.0562	-2.804	$C_5H_5N_5O$		MS^2[152]:152.0563(100),110.0349(15),135.0298(19)	Guanine	[3]	Other
11*	2.15	169.0142	169.0132	0.830	$C_7H_6O_5$	MS^2[169]:69.0331(8),81.0333(3),95.0126(2),125.0231(100),169.0140(4),107.572(2),97.028(8)		Gallic Acid	[2]	Phenols
12	2.65	166.0862	166.0858	-2.741	$C_9H_{11}NO_2$		MS^2[166]:120.0806(100),103.0543(9),93.0700(3),91.0544(1),79.0546(1)	L-phenylalanine	[2]	Amino acids

13	3.27	315.0721	315.0719	2.671	$C_{13}H_{16}O_9$	MS^2[315]:108.0203(100),152.0104(44),109.0284(25),153.0177(10)		protocatechuic acid 4-O-glucoside	[3]	Phenols
14	3.55	167.0349	167.0339	-2.955	$C_8H_8O_4$	MS^2[167]:167.0340(3),152.0104(100),123.0440(8),		Vanillic acid or its isomer	[5]	Phenols
15	3.85	153.0193	153.0182	0.097	$C_7H_6O_4$	MS^2[153]:81.0331(3),95.0122(1),108.0204(7),109.028(100),153.018(5)		Gentisic Acid or its isomer	[6]	Phenols
16	3.85	153.0193	153.0182	-0.230	$C_7H_6O_4$	MS^2[153]:109.0282(100),123.0438(54)		2,3-Dihydroxybenzoic acid	mzVault	Phenols
17	4.24	205.0971	205.0966	-2.702	$C_{11}H_{12}N_2O_2$		MS^2[205]:115.0542(1),117.0700(2),118.0650(41),132.0804(11),143.0728(4),146.0596(100),159.0911(10),170.0594(8),188.0700(11)	D-tryptophan	[2]	Amino acids
18	4.24	188.0706	188.0700	-2.845	$C_{11}H_9NO_2$		MS^2[188]:188.0650(100),115.0544(3),143.0728(13),144.0805(26),	3-Amino-2-naphthoic acid	[3]	organic acid
19	4.43	461.1664	461.1662	1.967	$C_{20}H_{30}O_{12}$	MS^2[461]:59.0124(42),71.0124(62),89.0229(19),95.0124(24),101.0231(8),113.0231(100),123.0440(5),135.0438(50),153.0546(3)		Verbasoside or its isomer	[7]	Phenols
20	4.85	487.1457	487.1454	1.771	$C_{21}H_{28}O_{13}$	MS^2[487]:179.0339(28),161.0233(27),135.0439(100)		1-O-Caffeioyl-(6-O-L rhamnopyr-anosyl)-glucopyranosid	mzVault	Phenylpropanoids
21	4.89	199.0600	199.0595	-2.813	$C_9H_{10}O_5$		MS^2[199]:95.0492(41),123.0442(6),125.0229(15),140.0463(100)	Syringic acid or its isomer	[1]	Phenols
22	5.29	153.0193	153.0182	-0.034	$C_7H_6O_4$	MS^2[153]:57.0330(1),69.0330(1),71.0122(1),81.0331(7),83.0124(1),95.0124(7),108.0203(100)		Gentisic Acid or its isomer	[6]	Phenols
23	5.46	421.1351	421.1348	0.686	$C_{17}H_{26}O_{12}$	MS^2[421]:113.0230(10),59.0125(100),68.9967(34),71.0124(42),89.0230(31),101.0231(42)		Lamiide	[8]	Terpenoids
24	5.67	153.1273	153.12697	-2.754	$C_{10}H_{16}O$		MS^2[153]:153.1270(22),107.0855(58),109.1012(43)	Citral	[3]	Terpenoids

25	5.73	461.1664	461.1664	2.293	$C_{20}H_{30}O_{12}$	MS^2[461]:61.9869(28),71.0125(11)		Verbasoside or its isomer	[7]	Phenols
26	5.99	431.1194	431.1191	1.711	$C_{18}H_{24}O_{12}$	MS^2[431]:59.0126(12),71.0123(7),101.0230(13),113.0232(8),135.0439(41),147.0442(21)		Asperulosidic acid	[9]	Terpenoids
27	6.00	387.1285	387.1272	-1.300	$C_{17}H_{22}O_{10}$		MS^2[387]:207.0645(74),193.0491(90),165.0543(49),95.0493(100),109.0649(21),81.0702(19),85.0288(20)	Methyl 7-formyl-1-[3,4,5-trihydroxy-6-(hydroxymethyl)oxan-2-yl]oxy-1,4a,5,7a-tetrahydrocyclopenta[c]pyran-4-carboxylate	mzVault	Terpenoids
28	6.10	403.1245	403.1242	1.866	$C_{17}H_{24}O_{11}$	MS^2[403]:59.0124(7),68.9967(100),71.0124(6),101.0230(98),135.0439(19),137.0231(9),143.0337(10),147.0438(7),163.0393(10),209.0444(9)		Theviridoside	[10]	Terpenoids
29	6.12	193.0495	193.0489	-2.876	$C_{10}H_8O_4$		MS^2[193]:79.0546(16),95.0493(100),137.0589(5),	Isoscopoletin	[11]	Phenylpropanoids
30	6.12	225.0757	225.0749	-3.465	$C_{11}H_{12}O_5$		MS^2[225]:95.0494(100),147.0436(14),175.0386(3),207.0647(20)	Sinapic acid	[11]	Phenylpropanoids
31	6.38	179.0702	179.0696	-3.522	$C_{10}H_{10}O_3$		MS^2[179]:133.0645(30),161.0587(8),105.0699(82)	2-Methoxycinnamic acid	[3]	Phenylpropanoids
32	6.38	135.0804	135.0800	-2.824	$C_9H_{10}O$		MS^2[135]:107.0855(66),135.0800(100),117.0697(73)	Cinnamyl Alcohol	[3]	Phenylpropanoids
33	6.54	195.0651	195.0645	-3.462	$C_{10}H_{10}O_4$		MS^2[195]:177.0541(34),149.0592(34),107.0490(10),95.0492(14),79.0546(19),	Ferulic acid	[11]	Phenylpropanoids
34	6.55	461.1664	461.1661	1.707	$C_{20}H_{30}O_{12}$	MS^2[461]:57.0332(11),59.0124(97),71.0124(84),83.0125(16),89.0233(7),95.0125(9),101.0230(76),11		Verbasoside or its isomer	[7]	Phenols

						3.0230(43),161.0448(5)				
35	6.69	167.0349	167.0339	-2.716	$C_8H_8O_4$	MS^2[167]:167.0334(5),152.0103(83),123.0439(65),		Vanillic acid or its isomer	[5]	Phenols
36	6.76	153.0193	153.0182	0.079	$C_7H_6O_4$	MS^2[153]:109.0281(100),153.0181(26)		Protocatechuic acid	[8]	Phenols
37	6.83	389.1089	389.1086	2.062	$C_{16}H_{22}O_{11}$	MS^2[389]:345.0672(5),181.0495(25),166.0262(100),151.0027(13)		Theveside	[12]	Terpenoids
38	6.93	639.1930	639.1929	0.658	$C_{29}H_{36}O_{16}$	MS^2[639]:161.0233(100),151.0390(24),179.0340(15)		β- hydroxyl -Verbascoside	mzVault	Phenylpropanoids
39	6.94	325.0917	325.0906	-3.457	$C_{15}H_{16}O_8$		MS^2[325]:163.0385(100),145.0280(30),135.0437(32),117.0334(12)	8-[3,4,5-Trihydroxy-6-(hydroxymethyl)oxan-2-yl]oxychromen-2-one	mzVault	Phenylpropanoids
40	6.95	197.0455	197.0447	1.320	$C_9H_{10}O_5$	MS^2[197]:89.0018(11),127.0024(3),138.0309(4),139.0024(3),166.9976(15),182.0209(11)		Syringic acid or its isomer	[1]	Phenols
41	7.24	461.1664	461.1651	-0.483	$C_{20}H_{30}O_{12}$	MS^2[461]:59.0124(72),71.0124(73),89.0229(34),95.0124(26),101.0230(73),113.0230(100),123.0435(7),131.0346(4),135.0438(55)		Verbasoside or its isomer	[7]	Phenols
42	7.34	741.2247	741.2251	2.030	$C_{33}H_{42}O_{19}$	MS^2[741]:161.0233(100),133.0283(30)		Fucatoside B	[8]	Phenylpropanoids
43	7.51	163.0400	163.0390	0.548	$C_9H_8O_3$	MS^2[163]:119.0489(100)		Coumaric acid	[12]	Phenylpropanoids
44	7.58	638.1852	638.1807	-5.243	$C_{29}H_{35}O_{16}$	MS^2[638]:161.0223(100)133.0282(34)		Hydroxy acteoside	[13]	Phenylpropanoids
45	8.23	652.2008	652.1959	-5.928	$C_{30}H_{37}O_{16}$	MS^2[652]:161.0233(100),133.0282(32)		Campneoside I	[14]	Phenylpropanoids
46	8.34	181.0488	181.0495	0.194	$C_9H_8O_4$		MS^2[181]:163.0384(100),145.0279(58),135.0436(97),117.0333	Caffeic acid or its isomer	[5]	Phenylpropanoids

							(42),107.0491(14),89.0388(25)			
47	8.34	325.0917	325.0904	-4.134	$C_{15}H_{16}O_8$		MS^2[325]:163.0384(100),145.0280(31),135.0437(34),117.0333(12),97.0285(15),69.0340(12)	3-Hydroxycoumarin glucoside	mzVault	Phenylpropanoids
48	8.34	471.1497	471.1481	-3.338	$C_{21}H_{26}O_{12}$		MS^2[471]:163.0384(100),145.0278(24),135.0437(22),85.0287(48),71.0496(50)	((2R,3R,4S,5R,6R)-6-(2-(3,4-dihydroxyphenyl)ethoxy)-3,5-dihydroxy-4-((2R,3R,4R,5R,6S)-3,4,5-trihydroxy-6-methyloxan-2-yl)oxyoxan-2-yl)methyl (E)-3-(3,4-dihydroxyphenyl)prop-2-enoate	mzVault	Phenylpropanoids
49	8.34	625.2126	625.2106	-3.210	$C_{29}H_{36}O_{15}$		MS^2[625]:163.0383(100),145.0279(16),135.0435(13),85.0287(14)	isomer of Verbascoside	mzVault	Phenylpropanoids
50	8.37	623.1981	623.1976	0.936	$C_{29}H_{36}O_{15}$	MS^2[623]:59.0124(6),71.0123(9),89.0229(4),95.0126(3),101.0230(2),113.0230(18),117.0331(1),123.0440(2),135.0439(16),161.0233(100)		isomer of Verbascoside	[12]	Phenylpropanoids
51	8.39	181.0495	181.0488	-3.564	$C_9H_8O_4$		MS^2[181]:135.0436(97),117.0333(42),89.0388(25),107.0491(14),111.0443(4),145.0279(58),163.0384(100)	Caffeic acid or its isomer	[6]	Phenylpropanoids
52	8.39	479.1547	479.1532	-3.189	$C_{23}H_{26}O_{11}$		MS^2[479]:163.0383(100),145.0279(23),135.0436(23),97.0286(6)	Calceolarioside A	mzVault	Phenylpropanoids
53	8.63	199.0600	199.0594	-3.064	$C_9H_{10}O_5$		MS^2[199]:95.0492(35),123.0437(5),125.0230(14),140.0463(100)	Syringic acid or its isomer	[1]	Phenols
54	8.97	147.0451	147.0440	-0.109	$C_9H_8O_2$	MS^2[147]:90.9699(3),103.9190(2),119.0490(3)		3-phenyl-2-Propenoic acid,	mzVault	Phenylpropanoids

55	9.09	621.1824	621.1823	0.684	$C_{29}H_{34}O_{15}$	MS^2[621]:161.0233(100),179.0341(29)		3’,4’-Dimethoxyluteolin-7-O-neo-hesperidin	mzVault	Flavonoids
56	9.13	471.1497	471.1481	-3.210	$C_{21}H_{26}O_{12}$		MS^2[471]:163.0384(100),145.0280(26),135.0437(25),85.0287(21),71.0496(18)	Umbelliferone 7-O-Rutinoside	mzVault	Phenylpropanoids
57	9.13	325.0917	325.0905	-3.826	$C_{15}H_{16}O_8$		MS^2[325]:163.0384(100),145.0280(30),135.0437(37),81.0338(17)	Mahaleboside	mzVault	Phenylpropanoids
58	9.13	625.2126	625.2104	-2.247	$C_{29}H_{36}O_{15}$	MS^2[623]:59.0124(8),71.0124(8),89.0230(3),95.0126(4),101.0232(2),113.0230(20),117.0333(1),123.0438(1),135.0439(19),161.0233(100),179.0340(4)	MS^2[625]:163.0384(100),145.0280(14),135.0436(13),85.0287(9)	isomer of Verbascoside	[12]	Phenylpropanoids
59	9.22	153.1273	153.1268	-0.512	$C_{10}H_{16}O$		MS^2[153]:153.1269(30),135.0801(30),97.0649(100),69.0704(49)	Camphor	[11]	Terpenoids
60	9.25	197.1172	197.1166	-2.896	$C_{11}H_{16}O_3$		MS^2[197]:133.1008(100)	Loliolide	[6]	Other
61*	9.93	137.0244	137.0232	-0.807	$C_7H_6O_3$	MS^2[137]:137.0232(14),93.0332(100)		Salicylic acid	[15]	Phenols
62	10.07	447.0921	447.0908	-2.925	$C_{21}H_{18}O_{11}$		MS^2[447]:271.0590(100)	6-(5,6-dihydroxy-4-oxo-2-phenylchromen-7-yl)oxy-3,4,5-trihydroxyoxane-2-carboxylic acid	[16]	Phenylpropanoids
63	10.11	636.2059	636.2028	-3.225	$C_{30}H_{37}O_{15}$	MS^2[636]:175.0391(49),160.0154(100)		Leucosceptoside A isomer 1	[17]	Phenylpropanoids
64	10.12	339.1074	339.1062	-3.491	$C_{16}H_{18}O_8$		MS^2[339]:177.0540(44),145.0279(100),117.0333(26),69.0340(10),97.0285(13)	(1R,3R,4R,5R)-3-[3-(3,4-dihydroxyphenyl)prop-2-enoyloxy]-4,5-dihydroxycyclohexane-1-carboxylic acid	mzVault	Phenylpropanoids

65*	10.13	187.0975	187.0966	0.879	$C_{9}H_{16}O_{4}$	MS^2[187]:57.0332(11),97.0645(32),123.0802(18),125.0959(100),187.0966(3)		Nonanedioic acid	[2]	organic acid
66	10.13	637.2137	637.2136	0.682	$C_{30}H_{38}O_{15}$	MS^2[637]:175.0391(49),135.0438(21),193.0501(6),160.0134(100),161.0231(15)		Cistanoside C	[12]	Phenylpropanoids
67	10.56	461.1089	461.1088	0.962	$C_{22}H_{22}O_{11}$	MS^2[461]:283.0246(100),255.0293(24)		Chrysoeriol-7-O-β-D-glucoside	[18]	Flavonoids
68	11.03	783.2717	783.2717	0.812	$C_{36}H_{48}O_{19}$	MS^2[783]:193.0491(5),175.0391(43),134.0361(29),160.0155(100)		Angoroside C	[19]	Phenylpropanoids
69	11.42	623.1981	623.1978	1.337	$C_{29}H_{36}O_{15}$	MS^2[623]:179.0339(8),161.0233(100),135.0438(21),123.0448(2),113.0231(20),95.0126(3),,89.0230(2)		isomer of Verbascoside	mzVault	Phenylpropanoids
70	11.73	151.1117	151.1112	-3.254	$C_{10}H_{14}O$		MS^2[151]:55.0548(16),67.0547(24),71.0496(11),79.0546(17),81.0702(100),91.0544(17),93.0700(51),95.0493(26),107.0854(18),109.0647(37),123.1165(33),133.1006(17)	Perillene	[11]	Other
71	12.26	325.0917	325.0906	-3.550	$C_{15}H_{16}O_{8}$		MS^2[325]:163.0384(100),145.0280(30),117.0334(12),135.0436(32)	7-[3,4,5-Trihydroxy-6-(hydroxymethyl)oxan-2-yl]oxychromen-2-one	mzVault	Phenylpropanoids
72	12.60	339.1074	339.1062	-3.403	$C_{16}H_{18}O_{8}$		MS^2[339]:177.0540(45),145.0280(100),117.0333(26),149.0592(11),97.0285(13),69.0340(11)	3-p-Coumaroylquinic acid	mzVault	Phenylpropanoids
73	12.64	651.2294	651.2295	1.848	$C_{31}H_{40}O_{15}$	MS^2[651]:193.0498(3),160.0155(100),175.0390(39)		Isomartynoside	[5]	Other
74	12.64	623.1981	623.1978	1.337	$C_{29}H_{36}O_{15}$	MS^2[623]:59.0125(7),71.0124(9),,89.0232(4),95.0126(3),101.0229(2),113.0230(18),123.0442(3),135.0441(29),161.0234(100),179.033		Isoacteoside	mzVault	Phenylpropanoids

						6(4)				
75	12.69	147.0451	147.0439	-0.449	$C_9H_8O_2$	MS^2[147]:87.9292(1),90.9700(2),103.9190(1)		2-Propenoic acid, 3-phenyl-	mzVault	Phenylpropanoids
76	13.00	161.0597	161.0591	-3.328	$C_{10}H_8O_2$		MS^2[161]:133.0644(38),103.0543(32),118.0411(26),161.0583(3),105.0699(100)	6-Methylcoumarin	[3]	Phenylpropanoids
77*	13.05	287.0550	287.0540	-3.256	$C_{15}H_{10}O_6$		MS^2[287]:287.0539(100),241.0485(2),171.0276(2),107.0488(1)	Kaempferol	[16]	Flavonoids
78*	13.06	285.0404	285.0402	3.107	$C_{15}H_{10}O_6$	MS^2[285]:65.0018(7),83.0124(4),107.0124(19),149.0232(17),150.9906(3),151.0025(43),185.0593(3),199.0390(17),217.0499(9),241.0506(4),243.0291(5),285.04010(58),133.0283(100),257.0757(2)		Luteolin	[20]	Flavonoids
79	13.15	317.0655	317.0646	-2.962	$C_{16}H_{12}O_7$		MS^2[317]:302.0410(100),186.0152(14),168.0048(10)	Nepetin	mzVault	Flavonoids
80*	13.19	317.0655	317.0647	-2.678	$C_{16}H_{12}O_7$		MS^2[317]:302.0410(100)	Isorhamnetin	[6]	Flavonoids
81	13.21	315.0510	315.0510	3.685	$C_{16}H_{12}O_7$	MS^2[315]:136.9868(48),300.0273(100)		Eupafolin	[8]	Flavonoids
82	13.43	191.0702	191.0697	-2.673	$C_{11}H_{10}O_3$		MS^2[191]:163.0749(25),149.0230(91),105.0699(23),91.0544(21),79.0546(17)	7-methoxy-4-methylcoumarin	[11]	Phenylpropanoids
83	13.54	511.1387	511.1376	-2.121	$C_{30}H_{22}O_8$		MS^2[511]:255.0645(2),155.0336(5),137.0229(100)	7-hydroxy-3-[7-hydroxy-2-(4-hydroxyphenyl)-4-oxo-3,4-dihydro-2H-1-benzopyran-3-yl]-2-(4-hydroxyphenyl)-3,4-dihydro-2H-1-benzopyran-4-one	mzVault	Flavonoids
84*	13.6	269.0455	269.0453	3.234	$C_{15}H_{10}O_5$	MS^2[269]:63.0225(5),65.0018(13)		Apigenin	[21]	Flavonoi

	5					,83.0124(7),107.0124(34),117.0332(100),149.0232(52),151.0025(68),159.0440(12),183.0439(7),197.0598(4),227.0346(6),241.0505(1),134.8387(2)				ds
85	13.67	327.2176	327.2174	2.474	$C_{18}H_{32}O_5$	MS^2[327]:57.0331(24),127.1117(13),171.1017(100),211.1333(58),229.1441(9)		9,12,13-Trihydroxy-10,15-octadecadienoic acid	mzVault	organic acid
86	13.73	301.0706	301.0695	-3.702	$C_{16}H_{12}O_6$		MS^2[301]:286.0462(100),186.0153(7),168.0048(13)	Hispidulin	mzVault	Phenylpropanoids
87*	13.76	299.0561	299.0556	2.259	$C_{16}H_{12}O_6$	MS^2[299]:284.0324(100),255.0298(4),256.0377(5),228.0423(9),136.9868(69),108.0201(2),92.1453(2)		Chrysoeriol	[20]	Flavonoids
88	13.77	301.0706	301.0695	-3.802	$C_{16}H_{12}O_6$		MS^2[301]:286.0462(100),258.0505(1),168.0048(14),121.0282(3),	Tectorigenin	[11]	Flavonoids
89	13.80	511.1387	511.1372	-3.001	$C_{30}H_{22}O_8$		MS^2[511]:255.0645(1),155.0332(5),137.0229(100),163.0382(3)	(2S,3S)-3-(2,4-dihydroxybenzoyl)-2,7-bis(4-hydroxyphenyl)-2,3,6,7-tetrahydrofuro[3,2-g]chromen-5-one	mzVault	Flavonoids
90	13.89	331.0812	331.0800	-3.652	$C_{17}H_{14}O_7$		MS^2[331]:316.0565(100),301.0331(20),186.0153(12),168.0047(10)	Jaceosidin	mzVault	Flavonoids
91	13.93	329.2333	329.2332	3.036	$C_{18}H_{34}O_5$	MS^2[329]:99.0802(43),127.1116(28),139.1116(40),171.1016(78),193.1228(9),211.1332(100),229.1440(31)		9,12,13-Trihydroxyoctadec-10-enoic acid	[22]	organic acid
92	13.94	331.0812	331.0800	-3.562	$C_{17}H_{14}O_7$		MS^2[331]:316.0565(100),301.0331(22),288.0612(2),273.0383(10),245.0437(4)	Iristetrigenin B	[11]	Flavonoids
93*	14.03	255.0662	255.0660	3.468	$C_{15}H_{12}O_4$	MS^2[255]:119.0490(100),135.0075(20)	MS^2[255]:81.0339(3),119.0490(15),137.0229(100),147.0436(31	Liquiritigenin	[5]	Flavonoids

							),257.0797(2)			
94*	14.14	671.1828	671.1769	-7.227	$C_{29}H_{36}O_{18}$	MS^2[671]:135.0075(84)		Verbascoside	[12]	Phenylpropanoids
95	14.20	287.0914	287.0904	-3.414	$C_{16}H_{14}O_5$		MS^2[287]:163.0385(10),145.0282(32),137.0229(100),117.0333(14),177.0540(13)	Homobutein	mzVault	Flavonoids
96	14.22	269.0455	269.0453	3.457	$C_{15}H_{10}O_5$	MS^2[269]:225.0549(17),227.0344(7),181.0651(7),201.0552(11),159.0443(7),		Genistein	mzVault	Flavonoids
97	14.30	511.1387	511.1373	-2.825	$C_{30}H_{22}O_8$		MS^2[511]:255.0641(20),239.0330(11),211.0383(18),137.0229(100),147.0436(87),119.0490(39)	1-[3-(2,4-Dihydroxybenzoyl)-6-hydroxy-2-(4-hydroxyphenyl)-2,3-dihydro-1-benzofuran-5-yl]-3-(4-hydroxyphenyl)prop-2-en-1-one	mzVault	Flavonoids
98	14.33	285.0404	285.0403	3.528	$C_{15}H_{10}O_6$	MS^2[285]:199.0395(18),175.0387(21),149.0233(18),133.0283(100),107.0125(19),57.0331(17),151.0027(39)		4H-1-Benzopyran-4-one, 2-(2,5-dihydroxyphenyl)-5,7-dihydroxy-	mzVault	Flavonoids
99	14.81	511.1387	511.1372	-3.001	$C_{30}H_{22}O_8$		MS^2[511]:137.0230(100),147.0436(90),119.0490(53),91.0544(15)	(2S,3R)-3-[5-[(E)-3-(2,4-dihydroxyphenyl)-3-oxoprop-1-enyl]-2-hydroxyphenyl]-7-hydroxy-2-(4-hydroxyphenyl)-2,3-dihydrochromen-4-one	mzVault	Flavonoids
100*	15.03	283.0611	283.0611	3.533	$C_{16}H_{12}O_5$	MS^2[283]:211.0388(1),268.0375(100),283.0611(2)，240.0422(4)	MS^2[285]:285.0747(100),270.0513(60),242.0564(58),135.0439	Calycosin	[2]	Flavonoids

							(1)			
101	15.09	511.1387	511.1373	-2.708	$C_{30}H_{22}O_8$		MS^2[511]:255.0637(1),155.0333(7),137.0229(100)	(E)-1-[3-(2,4-dihydroxybenzoyl)-4-hydroxy-2-(4-hydroxyphenyl)-2,3-dihydro-1-benzofuran-5-yl]-3-(4-hydroxyphenyl)prop-2-en-1-one	mzVault	Flavonoids
102	15.21	289.1798	289.1788	-3.427	$C_{18}H_{24}O_3$		MS^2[289]:91.0544(45),107.0491(24),55.0549(61),135.0800(28)	Psoracorylifol B or Isomer	[23]	Terpenoids
103	15.25	293.1758	293.1759	3.971	$C_{17}H_{26}O_4$	MS^2[293]:236.1050(14),221.1540(100),220.1462(66),204.1224(4)		Gingerol	[3]	Phenols
104	15.31	315.0863	315.0851	-3.823	$C_{17}H_{14}O_6$		MS^2[315]:300.0617(100),186.0153(16),168.0048(13)	5,7-dihydroxy-6-methoxy-2-(4-methoxyphenyl)chromen-4-one	mzVault	Flavonoids
105	15.32	487.3428	487.3428	2.050	$C_{30}H_{48}O_5$	MS^2[487]:469.3316(54),427.3232(32)		3,12,13-Trihydroxy-28-oleananoic acid; (3β,12β,13β)-form, 3-ketone	mzVault	Terpenoids
106	15.97	511.1387	511.1371	-3.119	$C_{30}H_{22}O_8$		MS^2[511]:155.0333(9),137.0229(100),111.0439(2)	3-[3-(2,4-Dihydroxybenzoyl)-2-(4-hydroxyphenyl)-2,3-dihydro-1-benzofuran-5-yl]-1-(2,4-dihydroxyphenyl)prop-2-en-1-one	mzVault	Flavonoids
107	16.16	487.3428	487.3428	2.050	$C_{30}H_{48}O_5$	MS^2[487]:469.3351(63),427.3243(70)		3,12,13-Trihydroxy-28-oleananoic acid; (3β,12β,13β)-form, 3-ketone	mzVault	Terpenoids

108	16.72	277.1798	277.1789	-3.107	$C_{17}H_{24}O_3$		MS^2[277]:137.0593(100)	Shogaol	[3]	Phenols
109*	16.99	469.3323	469.3319	1.499	$C_{30}H_{46}O_4$	MS^2[469]:469.3326(100),		Glycyrrhetinic acid	[3]	Terpenoids
110	17.77	455.3519	455.3504	-3.320	$C_{30}H_{46}O_3$		MS^2[455]:95.0857(100),121.1011(65),119.0854(77),109.1012(61),107.0855(91),81.0702(63)	Ursonic Acid	mzVault	Terpenoids
111	17.79	453.3374	453.3369	-3.584	$C_{30}H_{46}O_3$		MS^2[453]:67.0547(24),,81.0702(63),93.0700(49),95.0857(100),105.0698(43),107.0855(91),109.1012(61),135.1164(23),147.1163(36)	Wilforlide A	[3]	Terpenoids
112	18.28	295.2278	295.2274	2.400	$C_{18}H_{32}O_3$	MS^2[295]:171.1016(100),277.2167(46)		9,10-Epoxu-12(Z)-octade cenoicacid	[2]	organic acid
113	18.89	455.3519	455.3503	-3.518	$C_{30}H_{46}O_3$		MS^2[455]:145.1008(58),119.0854(100),93.0701(55),159.1162(54),133.1010(84),107.0855(87),95.0857(98)	(+)-Betulonic acid	mzVault	Terpenoids
114	18.89	437.3414	437.3399	-3.286	$C_{30}H_{44}O_2$		MS^2[437]:119.0854(64),109.1011(40),107.0855(64),95.0857(100),93.0701(31),81.0702(61)	(Z,6S)-2-methyl-6-[(10R,13S,14S,17S)-4,4,10,13,14-pentamethyl-3-oxo-1,2,5,6,9,11,12,15,16,17-decahydrocyclopenta[a]phenanthren-17-yl]hept-2-enoic acid	mzVault	Terpenoids
115	18.99	411.3621	411.3606	-1.483	$C_{29}H_{46}O$		MS^2[411]:159.1164(65),133.1010(43),107.0856(73),81.0702(100),55.0549(54)	Stigmasta-5,24(28)-dien-3-one	mzVault	Terpenoids
116	18.99	429.3727	429.3712	-3.394	$C_{29}H_{48}O_2$		MS^2[427]:161.1329(20),135.1168(20),109.1011(68),83.0858(100),57.0705(63)	(3S,8S,9S,10R,13R,14S,17R)-3-hydroperoxy-10,13-dimethyl-17-[(Z,2R)-5-propan-2-ylhept-5-en-2-yl]-	mzVault	Terpenoids

								2,3,4,7,8,9,11,12,14,15,16,17-dodecahydro-1H-cyclopenta[a]phenanthrene		
117	22.31	277.2173	277.2170	3.006	$C_{18}H_{30}O_2$	MS^2[277]:54.0441(37)		Linolenic acid	mzVault	Other
118	22.33	439.3570	439.3555	-3.544	$C_{30}H_{46}O_2$		MS^2[439]:119.0853(62),109.1011(64),133.1008(51),121.1010(55),95.0857(100),107.0855(52),81.0702(46)	18-hydroxy-1,2,5,8,15,19,19-heptamethylpentacyclo[12.8.0.0<2,11>.0<5,10>.0<15,20>]docos-11-ene-8-carboxylic acid	mzVault	Terpenoids
119	22.69	439.3570	439.3555	-3.476	$C_{30}H_{46}O_2$		MS^2[439]:95.0857(100),81.0702(73),67.0547(18),109.1012(30),137.1321(23),123.1164(18)	2-(3a,5a,5b,8,8,11a-hexamethyl-9-oxo-2,3,4,5,6,7,7a,10,11,11b,12,13,13a,13b-tetradecahydro-1H-cyclopenta[a]chrysen-1-yl)prop-2-enal	mzVault	Terpenoids
120*	22.72	457.3676	457.3652	-5.164	$C_{30}H_{48}O_3$		MS^2[457]:133.009(59),119.1011(100),189.1631	Betulinic acid	[15]	Terpenoids
121*	23.21	455.3530	455.3526	0.241	$C_{30}H_{48}O_3$	MS^2[455]:455.3530(100),407.3330(6)		Ursolic acid	[1]	Terpenoids
122	23.22	439.3570	439.3555	-3.476	$C_{30}H_{46}O_2$		MS^2[439]:203.1788(65),147.1163(55),121.1009(65),95.0857(100),119.0853(76),109.1011(56)	3-Hydroxyolean-12-en-28-oic acid	mzVault	Terpenoids
123	23.22	457.3676	457.3660	-3.418	$C_{30}H_{48}O_3$		MS^2[457]:95.0857(100),109.1012(53),107.0857(70),121.1008(49),163.1634(48),133.1012(34),149.1323(32)	(1S,2R,4aR,6aR,6aS,6bR,8aR,10R,12aR,14bR)-10-hydroxy-1,2,6a,6b,9,9,12a-heptamethyl-	mzVault	Terpenoids

								2,3,4,5,6,6a,7,8,8a,10,11,12,13,14b-tetradecahydro-1H-picene-4a-carboxylic acid		
124	23.43	439.3570	439.3555	-3.476	$C_{30}H_{46}O_2$		MS^2[439]:218.1786(47),119.0854(32),109.1012(40),95.0857(100),121.1166(33),81.0703(74)	Betulonal	mzVault	Terpenoids
125	23.83	279.2329	279.2326	2.913	$C_{18}H_{32}O_2$	MS^2[279]:134.8937(100)		Linoleic acid	[3]	organic acid
126	25.17	255.2329	255.2325	2.755	$C_{16}H_{32}O_2$	MS^2[255]:255.2331(23)		Palmitic Acid	[15]	organic acid

Note: The label "*" represents that the compound was compared with the reference compound.

Table S2. Detailed information of the 15 reference standards

Compound Name	Formula	Lot Number	Supplier
Gallic acid	$C_7H_6O_5$	LW7525000	Shandong West Asia Chemical Co., Ltd
Salicylic acid	$C_7H_6O_3$	VO0525000	Shandong West Asia Chemical Co., Ltd
Calycosin	$C_{16}H_{12}O_5$	AF200211603	Chengdu Alfa Biotechnology Co. Ltd
Verbascoside	$C_{29}H_{36}O_{15}$	AZ22011365	Chengdu Alfa Biotechnology Co. Ltd
Liquiritigenin	$C_{15}H_{12}O_4$	AB0563-0020	Chengdu Alfa Biotechnology Co. Ltd
Luteolin	$C_{15}H_{10}O_6$	M-007-190422	Chengdu Refine Bio-Tech Co., Ltd
Kaempferol	$C_{15}H_{10}O_6$	S-014-171216	Chengdu Refine Bio-Tech Co., Ltd
Betulinic acid	$C_{30}H_{48}O_3$	RDD-B01502009002	Chengdu Refine Bio-Tech Co., Ltd
Chrysoeriol	$C_{16}H_{12}O_6$	HR1122W3	Baoji Chenguang Biotechnology Co., Ltd
Malic acid	$C_4H_6O_5$	M105695	Aladdin
Ursolic acid	$C_{30}H_{48}O_3$	MUST-14102905	Chengdu Monst Biotech Co., Ltd
Isorhamnetin	$C_{16}H_{12}O_7$	Y-039-181103	Chengdu Refine Bio-Tech Co., Ltd
Glycyrrhetinic Acid	$C_{30}H_{46}O_4$	AF21020353	Chengdu Alfa Biotechnology Co. Ltd
Apigenin	$C_{15}H_{10}O_5$	PCS0751	Chengdu Zhibiao Huachun Biotechnology Co., Ltd.
Nonanedioic acid	$C_9H_{16}O_4$	CM1980000	Shandong West Asia Chemical Co., Ltd

Table S3. Abbreviation list

Abbreviation	Full English Name
BPI	Base Peak Ion
ESI	Electrospray Ionization
FBMN	Feature-Based Molecular Networking

GNPS	Global Natural Products Social Molecular Networking
MRSA	Methicillin-Resistant Staphylococcus aureus
MSI	Metabolomics Standards Initiative
NMR	Nuclear Magnetic Resonance
RDA	Retro-Diels–Alder
SIRIUS	Spectral Inference of Unknowns via Rational Isotope and Fragment Utilization
UHPLC-Q-Exactive-Orbitrap MS	Ultra-High Performance Liquid Chromatography Coupled with Quadrupole-Exactive-Orbitrap Mass Spectrometry
ZODIAC	Zero-defect Organic compound Determination via Isotope And Compound clustering

References

1. Sun, J. W.; Li, G. T.; Chen, B.; Zhang, R.; Lu, Q.; Wan, J.; Sun, Y. D.; Cao, Y. M.; Hu, L. H.; Wang, X. C.; Gao, H. Q., Analysis of Components and Evaluation of Anti-inflammatory Activity of Zhejiang Red Camellia Based on UPLC-Q-TOF-MS/MS Combined with Molecular Networking Technology. *Chinese herbal medicine* **2025,** 56, (12), 4187-4205.
2. Xiong, H.; Li, N.; Yu, Y.; Liu, Z.; Li, Z.; Zhao, L., Comprehensive Characterization and Identification of Chemical Constituents of Xiangsha Pingwei Pills by UPLC-Q-TOF-MS. *J AOAC Int* **2023,** 106, (4), 1017-1036.
3. He, T. Y.; Wang, L.; Li, L.; Su, L. L.; Xie, H.; Zhao, X. L.; Mao, J.; Guo, Y.; Hou, J. C.; Mao, C. Q.; Lu, T. L.; Mei, X., Identification of chemical components based on UPLC-Q-TOF-MS/MS and network pharmacology of Zhuru Decoction. *China Journal of Chinese Materia Medica* **2022,** 47, (19), 5235-5245.
4. Wang, Y.; Hu, W. D.; Hong, L. L.; Xu, X. Y.; Wang, H. D.; Wang, X. Y.; Yang, W. Z.; Gao, X. M., Characterization and identification of chemical components from Euodiae Fructus based on UHPLC-Q-TOF-MS. *China Journal of Chinese Materia Medica* **2024,** 49, (11), 2953-2964.
5. Li, H.; Zhao, H.; Yang, Y.; Qi, D.; Cheng, X.; Wang, J., Identification of Chemical Components of Qi-Fu-Yin and Its Prototype Components and Metabolites in Rat Plasma and Cerebrospinal Fluid via UPLC-Q-TOF-MS. *Evid Based Complement Alternat Med* **2021,** 2021, 1995766.
6. Duan, H.; Wang, G. C.; Khan, G. J.; Su, X. H.; Guo, S. L.; Niu, Y. M.; Cao, W. G.; Wang, W. T.; Zhai, K. F., Identification and characterization of potential antioxidant components in Isodon amethystoides (Benth.) Hara tea leaves by UPLC-LTQ-Orbitrap-MS. *Food Chem Toxicol* **2021,** 148, 111961.
7. Xue, S.; Fu, Y.; Sun, X.; Chen, S., Changes in the Chemical Components of Processed Rehmanniae Radix Distillate during Different Steaming Times. *Evid Based Complement Alternat Med* **2022,** 2022, 3382333.

8. Darwish, R. S.; El-Banna, A. A.; Ghareeb, D. A.; El-Hosseny, M. F.; Seadawy, M. G.; Dawood, H. M., Chemical profiling and unraveling of anti-COVID-19 biomarkers of red sage (Lantana camara L.) cultivars using UPLC-MS/MS coupled to chemometric analysis, in vitro study and molecular docking. *J Ethnopharmacol* **2022,** 291, 115038.
9. Barupal, D. K.; Fiehn, O., Generating the Blood Exposome Database Using a Comprehensive Text Mining and Database Fusion Approach. *Environ Health Perspect* **2019,** 127, (9), 97008.
10. Pan, W. D.; Li, Y. J.; Mai, L. T.; Ohtani, K.; Kasai, R.; Tanaka, O., [Studies on chemical constituents of the roots of Lantana camara]. *Yao Xue Xue Bao* **1992,** 27, (7), 515-21.
11. Su, B.; Tian, J.; Wang, K.; Yang, W.; Ning, J.; Liang, Y.; Liu, Y.; Li, Y.; Zheng, G., Qualitative and Quantitative Analyses of the Chemical Components of Peels from Different Pomelo Cultivars (Citrus grandis [L.] Osbeck) Based on Gas Chromatography-Mass Spectrometry, Ultraperformance Liquid Chromatography-Q-Exactive Orbitrap-MS, and High-Performance Liquid Chromatography-Photodiode Array Detection. *ACS Omega* **2023,** 8, (7), 6253-6267.
12. El-Din, M. I. G.; Fahmy, N. M.; Wu, F.; Salem, M. M.; Khattab, O. M.; El-Seedi, H. R.; Korinek, M.; Hwang, T. L.; Osman, A. K.; El-Shazly, M.; Fayez, S., Comparative LC-LTQ-MS-MS Analysis of the Leaf Extracts of Lantana camara and Lantana montevidensis Growing in Egypt with Insights into Their Antioxidant, Anti-Inflammatory, and Cytotoxic Activities. *Plants (Basel)* **2022,** 11, (13).
13. Yamada, P.; Iijima, R.; Han, J.; Shigemori, H.; Yokota, S.; Isoda, H., Inhibitory effect of acteoside isolated from Cistanche tubulosa on chemical mediator release and inflammatory cytokine production by RBL-2H3 and KU812 cells. *Planta Med* **2010,** 76, (14), 1512-8.
14. Wu, H. F.; Zhu, Y. D.; Zhang, L. J.; Zou, Q. Y.; Chen, L.; Shen, T.; Wang, X. F.; Ma, G. X.; Hu, B. R.; Hu, W. C.; Xu, X. D., A new phenylethanoid glycoside from Incarvillea compacta. *J Asian Nat Prod Res* **2016,** 18, (6), 596-602.
15. Li, X.; Sdiri, M.; Peng, J.; Xie, Y.; Yang, B. B., Identification and characterization of chemical components in the bioactive fractions of Cynomorium coccineum that possess anticancer activity. *Int J Biol Sci* **2020,** 16, (1), 61-73.
16. Zhou, M.; Huo, J.; Wang, C.; Wang, W., UPLC/Q-TOF MS Screening and Identification of Antibacterial Compounds in Forsythia suspensa (Thunb.) Vahl Leaves. *Front Pharmacol* **2021,** 12, 704260.
17. Ersöz, T.; Alipieva, K. I.; Yalçin, F. N.; Akbay, P.; Handjieva, N.; Dönmez, A. A.; Popov, S.; Caliş, I., Physocalycoside, a new phenylethanoid glycoside from Phlomis physocalyx Hub.-Mor. *Z Naturforsch C J Biosci* **2003,** 58, (7-8), 471-6.
18. Zhang, J.; Pang, D. R.; Huang, Z.; Huo, H. X.; Li, Y. T.; Zheng, J.; Zhang, Q.; Zhao, Y. F.; Tu, P. F.; Li, J., Flavonoids from whole plants of Lagopsis supina. *Zhongguo Zhong Yao Za Zhi* **2015,** 40, (16), 3224-8.
19. de Santos, J.; Díaz Lanza, A. M.; Fernández, L.; Rumbero, A., Isoangoroside C, a phenylpropanoid glycoside from Scrophularia scorodonia roots. *Z Naturforsch C J Biosci* **2000,** 55, (5-6), 333-6.
20. Valério, G. B.; Godinho, C. C.; Freitas, T. R.; Santiago, M. B.; Martins, D. O. S.; Jardim, A. C. G.; Gobbo-Neto, L.; Martins, C. H. G.; Cunha, L. C. S.; Pilon, A. C.; Coqueiro, A.; Pivatto, M.; Danuello, A., Dereplication of Lantana trifolia L. leaves and fruits by UFLC-DAD-(+)-ESI-MS/MS and its antifungal and cytotoxic activities. *Metabolomics* **2023,** 19, (8), 68.

21. Leite, P. M.; Miranda, A. P. N.; Gomes, I.; Rodrigues, M. L.; Camargos, L. M.; Amorim, J. M.; Duarte, R. C. F.; Faraco, A. A. G.; Carvalho, M. G.; Castilho, R. O., Antithrombotic potential of Lippia alba: A mechanistic approach. *J Ethnopharmacol* **2023,** 301, 115744.
22. Doi, M.; Morita, N.; Okuzawa, T.; Ohgiya, S.; Okamoto, D.; Sato, K.; Ito, Y.; Matsuura, H.; Hashidoko, Y., Pinellic Acid Isolated from Quercetin-rich Onions has a Peroxisome Proliferator-Activated Receptor-Alpha/Gamma (PPAR-α/γ) Transactivation Activity. *Planta Med* **2022,** 88, (6), 440-446.
23. Hu, W. D.; Wang, S. Y.; Xu, A. C.; Wang, X. Y.; Gao, X. M.; Yang, W. Z., Characterization and identification of chemical components in traditional Chinese medicine Psoraleae Fructus based on UHPLC-Q-TOF-MS. *China Journal of Chinese Materia Medica* **2023,** 48, (11), 2989-2999.
